# The role of positive feedback loop between LINC00862 and RBM47 in hepatocellular carcinoma suppression

**DOI:** 10.1016/j.gendis.2025.101763

**Published:** 2025-07-09

**Authors:** Tao Guo, Yingying Jiang, Shunshun Zhu, Min Shi, Linying Sun, Juan Feng, Zhen Li, Cheng Gong

**Affiliations:** a Department of Pathophysiology, School of Basic Medical Sciences, Shandong Second Medical University, Weifang, Shandong 261053, China; b School of Stomatology, Shandong Second Medical University, Weifang, Shandong 261053, China; c Department of Functional Laboratory, School of Basic Medical Sciences, Shandong Second Medical University, Weifang, Shandong 261053, China; d School of Public Health, Wuhan University, Wuhan, Hubei 430072, China; e Department of Hepatobiliary and Pancreatic Surgery, Zhongnan Hospital of Wuhan University, Wuhan, Hubei 430071, China

**Keywords:** Hepatocellular carcinoma, Hoogsteen pair, LINC00862, lncRNA, RBM47

## Abstract

While the dynamic interaction between long non-coding RNA (lncRNA) and RNA binding proteins is widely recognized as pivotal in the regulation of hepatocellular carcinoma (HCC), the precise underlying mechanisms and networks governing their effects remain elusive. Here, we have uncovered LINC00862, a novel lncRNA that exhibits pronounced down-regulation in HCC tissues and whose expression levels are linked positively to favorable HCC outcomes, as a function of tumor stage and size. Through functional assays, we have established the anti-tumor effects of LINC00862 on HCC processes such as proliferation, invasion, metastasis, and growth *in vitro* and *in vivo*. Mechanistically, RNA sequencing and quantitative proteomics analyses have revealed that LINC00862's downstream target effector in HCC cells is RBM47. Our further experimentation strongly supports RBM47 as a central mediator of LINC00862's tumor-suppressive effects. Furthermore, our study elucidates the ability of LINC00862 to engage in Hoogsteen pairing interaction with the RBM47 promoter, while simultaneously recruiting the transcription factor CHD5 to elicit RBM47 transcriptional activation, ultimately resulting in transcriptional up-regulation and its consequential expression in hepatoma cells. It is intriguing to note that we also discovered that RBM47 could act as a transcription factor, positively regulating LINC00862 expression. Our identification of a positive feedback loop involving LINC00862 and RBM47 expands our comprehension of the intricate regulatory network that shapes HCC pathogenesis. LINC00862 represents a promising molecular marker for HCC diagnosis and therapeutics.

## Introduction

Hepatocellular carcinoma (HCC), the most compelling liver malignancy, substantiates the predominant clinical tendency of liver cancers and ranks among the most diagnosed tumors globally.^1^ HCC inflicts over 750,000 individuals annually globally, and most HCC patients are subject to a dismal prognosis, with a five-year overall survival rate falling below 50%.^2^^,^^3^ Nevertheless, the array of options for HCC therapy is limited, and the molecular mechanisms underpinning HCC are still inadequately explored to date.^4^

The past decade has witnessed significant advancements in deep sequencing technology, igniting a growing interest in functional long noncoding RNAs (lncRNAs).^5^ Extensive research into HCC has illuminated the dysregulated function of lncRNAs during the onset and progression of HCC, with these dysfunctions being associated with patient metastasis, disease recurrence, and impaired clinical outcomes, thus highlighting dysregulated functional lncRNAs as potential molecular markers for HCC growth and metastasis.^6^ lncRNAs may modulate HCC tumor behavior and tissue phenotype by altering their own functional properties, thereby serving as either promoters or suppressors for HCC. Mechanistically, lncRNAs can interact with various molecular species within the cellular environment, thereby playing a crucial role in shaping cellular fate.^7^ Furthermore, the specific subcellular localization of lncRNAs is a determinant of the distinct molecular mechanisms in which they engage and exert regulatory effects.^8^

On the other hand, RNA-binding motif (RBM) proteins are part of the RNA-binding protein (RBP) family, playing vital roles in the regulation of RNA editing, alternative splicing, transcript stability, and mRNA translation.^9^^,^^10^ RNA-binding motif protein 47 (RBM47) is an RBM member that acts as an RBP, binding directly to RNA to regulate alternative splicing.^11^ Recent findings have revealed that in addition to its role as an RBP, RBM47 performs crucial transcriptional regulatory functions.^12^ In our previous study, we observed that RBM47 significantly stifled HCC progression and that it could concurrently bind to DNA and RNA, functioning both as a transcription factor and an RBP.^13^ Therefore, RBM47 may have undiscovered roles as a multifunctional tumor suppressor in HCC.

As a DNA-binding protein, chromodomain helicase DNA-binding protein 5 (CHD5) functions as an ATP-dependent chromatin remodeler, capable of modulating target gene expression at the transcriptional level through its diverse roles, thereby facilitating neuronal differentiation.^14^ Consequently, CHD5 plays a particularly crucial role in the regulation of cellular proliferation and differentiation during neuronal development.^15^ In neurogenic tumors, such as high-risk neuroblastoma, CHD5 frequently exhibits heterozygous deletions.^16^ Subsequent research has further established CHD5 as a tumor suppressor in neuroblastoma.^17^ Additionally, in various other malignancies, CHD5 can also exert anti-cancer effects, either through direct DNA binding or by functioning as a transcription factor.^18^

Several studies employing sequencing technology have already identified a plethora of dysregulated lncRNAs that could serve as potential molecular targets or signatures for HCC.^19^^,^^20^ In a recent multi-sample clinical tissue sequencing study conducted by Yang et al, we noticed that long intergenic non-protein coding RNA 862 (LINC00862), an uncharacterized functional lncRNA, displayed aberrant expression in HCC tissue.^21^ Furthermore, based on the insights gained from our preliminary experiments, we identified a regulatory network involving LINC00862, CHD5, and RBM47, which significantly influences the progression of HCC. This study aims to elucidate the functional and molecular mechanisms of LINC00862 and its interaction with CHD5/RBM47 in regulating HCC progression, with the ambition of furnishing novel molecular targets and theoretical bases for HCC diagnosis and treatment.

## Materials and methods

### Tissue samples and clinical data

Paired clinical samples were obtained from patients undergoing hepatic surgical resection, consisting of HCC and corresponding paracancerous tissue from the same patient. The following inclusion criteria were used for data analysis: i) a diagnosis of primary HCC; ii) adult patients; iii) samples obtained from surgical resection with sufficient paired adjacent tissue; iv) no suspicion of intraoperative tumor spreading. Patients with the following conditions were excluded: i) a history of any cancer, metastatic hepatic cancer, or combination with other tumors; ii) hospitalized death; iii) incomplete clinical data or refusal of follow-up; iv) preoperative trans-arterial chemoembolization or other chemotherapy; v) tissues without adequate volume. Samples were immediately stored in liquid nitrogen on the operating table for temporary transportation, and the remaining samples were sufficient for pathological diagnosis. All included tissues were long-term preserved at −80 °C until use. Preserved tissues were analyzed for the level of LINC00862, but patient anonymity was ensured through data analysis based on unique identification numbers and by maintaining the confidentiality of clinical data.

### Cell culture and transfection

For the cultivation of hepatoma cells, a complete culture medium was utilized, incorporating Dulbecco's modified Eagle medium (Hyclone, SH30022.01B) with 10% fetal bovine serum (Lonsera, S711–001S) at 37 °C with 5% CO_2_. The HepG2, Huh7, SK-HEP-1, and Hep3B cell lines were obtained from Pricella Cell Bank (Procell Life Science & Technology Co., Ltd.), while the HCCLM3 cells were purchased from Cellverse (iCell Bioscience Inc.). The smart silencer (SS) targeting LINC00862 was generated by RiboBio Biotechnology. To realize knockdown of RBM47 and CHD5, small interfering RNAs (siRNAs) were ordered from Duolaimi Biotechnology. The full length or sequence-deletion of LINC00862 was also utilized for overexpression by loading it onto the pcDNA3.1 vector. To achieve overexpression of RBM47, the pTT5 plasmid vector was fused 3 × Flag tag amino acids (DYKDDDDK) with RBM47. Transfection of the SS and siRNAs was conducted at a 40 nM concentration utilizing Lipofectamine 2000 (ThermoFisher, 11668019), with the protocol followed per the manufacturer's instructions.

### *In vivo* and *in vitro* experiments

Further details on the matter are elaborated upon in the supplementary materials.

### Statistical analysis

For clinical data analysis, dichotomous variables were evaluated using the *χ*^2^ test or Fisher's exact test, while continuous variables were compared using paired or unpaired student's *t*-test. Survival rates were quantitatively estimated using Kaplan–Meier analysis, which was further evaluated using the log-rank test. Univariate and multivariate analyses based on Cox regression modeling were performed according to related clinical parameters. Binary relevance was evaluated using Pearson's analysis. A rigorous statistical significance level of *P* < 0.05 was employed, with all analyses carried out using SPSS 22.0 software package (IBM).

## Results

### LINC00862 has demonstrated clinical significance in both its expression levels and prognostic implications

**Figure 1:**
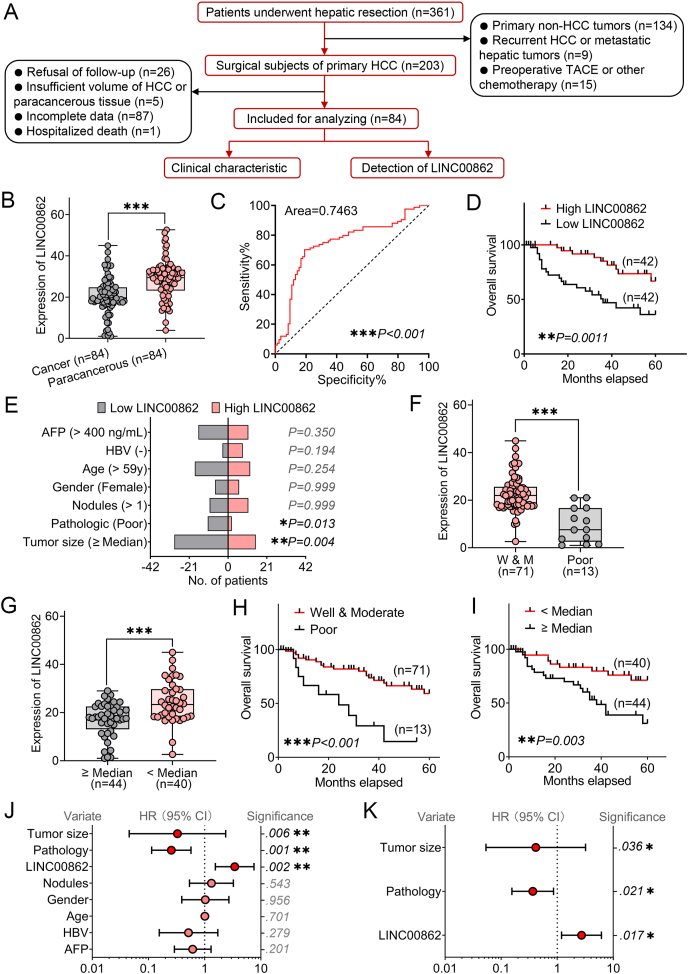
The clinical features of LINC00862 in hepatocellular carcinoma (HCC). **(A)** The schematic representation of the clinical sample inclusion process. **(B)** The quantitative reverse transcription PCR analysis displayed the expression of LINC00862 in 84 HCC and the corresponding paracancerous tissues. **(C)** The receiver operating characteristic curve of LINC00862 distinguished HCC tissues from paracancerous tissues. **(D)** The overall survival of HCC patients was plotted regarding LINC00862 expression. **(E)** The clinical characteristics of the high and low LINC00862 groups were compared. **(F)** The level of LINC00862 expression was assessed in 84 HCC tissues with varying pathological grades. **(G)** The level of LINC00862 expression was also examined in 84 HCC tissues with different tumor sizes. **(H, I)** The tumor pathology (H) and the tumor size (I) were analyzed and plotted on the Kaplan–Meier curve to show the overall survival. **(J)** The univariate analysis using the Cox proportional hazard regression model was performed to assess the overall survival of the included HCC subjects. **(K)** A multivariate analysis was performed based on the Cox regression model, which included tumor size, tumor pathology, and LINC00862 expression. The asterisks designate the statistical significance of the results as determined by the corresponding *p*-value (∗*P* < 0.05, ∗∗*P* < 0.01, and ∗∗∗*P* < 0.001). TACE, trans-arterial chemoembolization.

Following a careful selection process anchored on rigorous inclusion and exclusion criteria, we included 84 patients diagnosed with HCC and their paired paracancerous tissue from an initial pool of 361 candidates (Fig. 1A). Our analysis revealed a significant down-regulation of LINC00862 in HCC tissues compared with its paracancerous counterparts by quantitative reverse transcription PCR (qRT-PCR) (Table S1; Fig. 1B). Notably, the receiver operating characteristic curve analysis indicated that the optimal cut-off value for the fold change of LINC00862 was determined to be 24.62, yielding a corresponding sensitivity of 72.62% and specificity of 75%. These results imply a potential diagnostic efficacy for differentially expressed LINC00862 (area = 0.7463) (Fig. 1C). We further generated overall survival curves that demonstrated a favorable prognosis for patients with high levels of LINC00862 expression (Fig. 1D). We also observed that patients with low LINC00862 expression had poorly classified pathological characteristics and a higher incidence of tumors larger than the median size (Fig. 1E). We then conducted additional analyses to better understand the relationship between LINC00862, pathological grading, and tumor size. Here, we found LINC00862 to be significantly down-regulated in poorly classified and large tumors (Fig. 1F, G). Pathological type and tumor size were both factors that greatly influenced patient prognosis (Fig. 1H, I). Hence, LINC00862 expression levels hold the potential to relate to these factors as well. We leveraged Cox regression modeling in a univariate analysis to verify the association of LINC00862 expression levels, tumor size, and pathological grading with patient prognosis (Fig. 1J). Our multivariate analysis solidified these findings, as we identified LINC00862 expression levels, pathological type, and tumor size as independent prognostic factors for HCC patients (Fig. 1K). Therefore, evaluating LINC00862 expression levels has the potential to serve as a valuable tool for clinical diagnosis and facilitating the prediction of patient prognosis.

### LINC00862 exerts inhibitory effects on HCC progression both *in vitro* and *in vivo*

Considering the clinical significance of LINC00862, we postulated that it could hold a significant regulatory role in HCC. We began by examining LINC00862 expression in various hepatoma cell lines before proceeding to knock down LINC00862 in Huh7 cells and overexpress it in HCCLM3 cells to evaluate its impact on HCC's tumor behavior *in vitro* (Fig. S1A–C). To elucidate the precise mechanism underlying LINC00862's anti-cancer effect in HCC, we initially employed the lncATLAS database (https://lncatlas.crg.eu/) to predict the subcellular localization of LINC00862.^22^ Our results indicated predominant localization in the nuclei of hepatoma cells (Fig. S1D). We subsequently corroborated this assertion via RNA fluorescent *in situ* hybridization assays in Huh7 and HCCLM3 cells, confirming nuclear predominance of LINC00862 (Fig. S1E).

**Figure 2:**
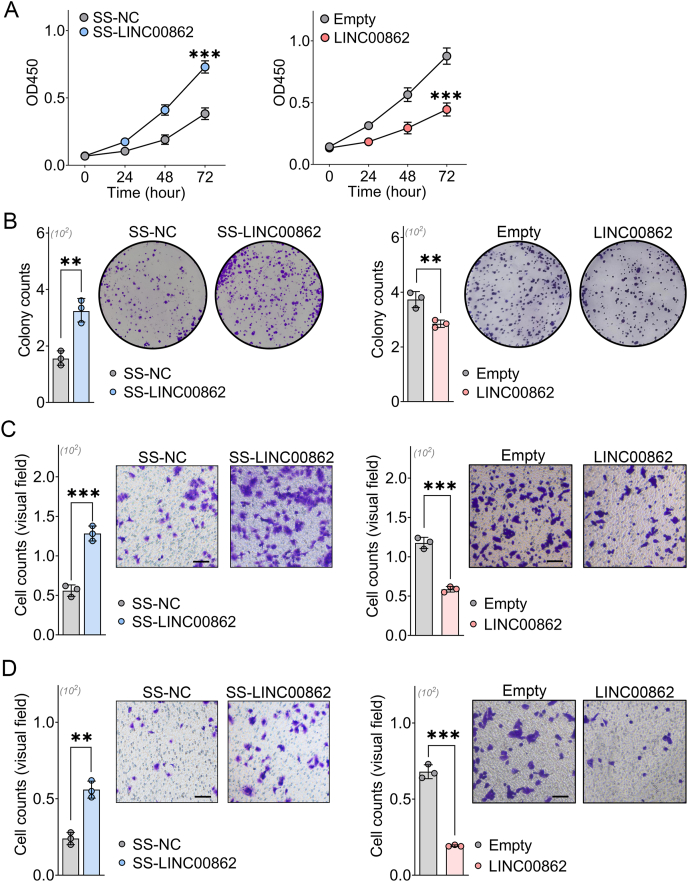
The effect of LINC00862 on oncogenic behaviors *in vitro* was evaluated through silencing LINC00862 in Huh7 cells and overexpressing it in HCCLM3 cells. **(A)** Using the CCK8 assay, the proliferation rate of hepatoma cells was evaluated and plotted to generate proliferation curves. **(B)** The colony formation assay was conducted to examine the colonizing ability of hepatoma cells, with representative images taken to visualize the cell clustering and adhesion process. **(C, D)** Migration (C) and invasion (D) abilities were evaluated through a transwell assay, with the microscopical visual fields of hepatoma cells being presented as a means of analysis. Black bar = 100 μm ∗∗*P* < 0.01 and ∗∗∗*P* < 0.001. All experiments were performed in duplicate. Mean and standard deviation are depicted from three independent experiments. SS, smart silencer.

**Figure 3:**
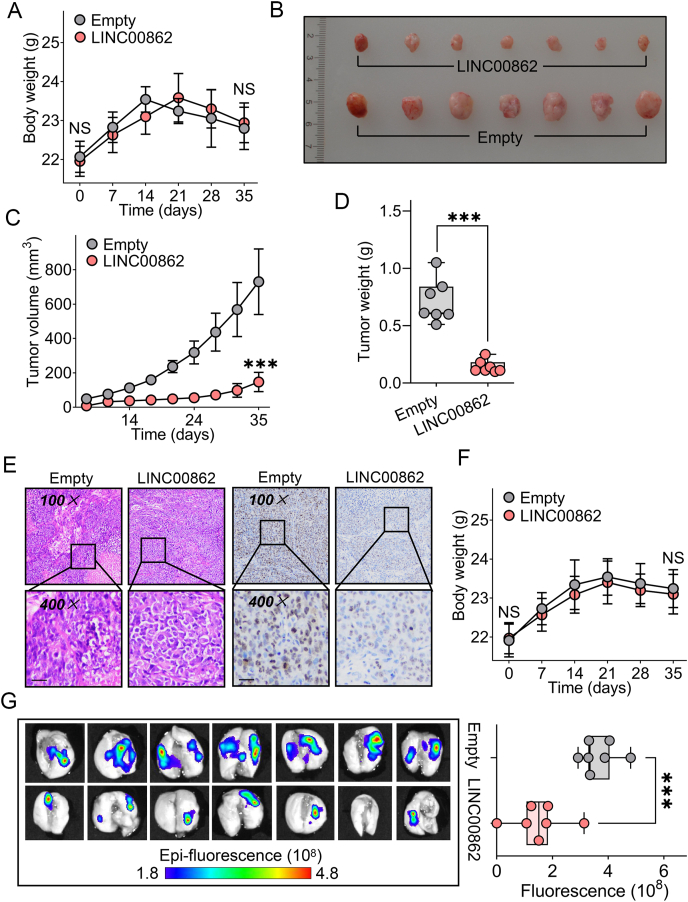
The inhibitory efficacy of LINC00862 on oncogenic behaviors *in vivo*. **(A)** Documentation of the weights of nude mice from the moment of subcutaneous tumor implantation up to the point of sacrifice (*n* = 7 per group). **(B)** Representative images of mouse xenograft tumors implanted with HCCLM3 cells that had been stably transfected with LINC00862 (*n* = 7 per group). **(C, D)** The growth curves (C) and tumor weight (D) were assessed following subcutaneous tumorigenesis (*n* = 7 per group). **(E)** Microscopical images of hematoxylin-eosin staining (left) and immunohistochemistry staining of Ki-67 (right) were analyzed in xenograft tumors with stably transfected LINC00862. Black bar = 50 μm. **(F)** Record of nude mice weights in the LINC00862 and empty groups following establishment of the pulmonary metastasis model (*n* = 7 per group). **(G)***In vivo* imaging of lung samples (left) and fluorescence quantitation (right) were assessed after the tail-vein injection of HCCLM3 cells that were stably transduced with LINC00862 (*n* = 7 per group). NS, no significance. ∗∗∗*P* < 0.001.

Our cell proliferation assays demonstrated that the knockdown of LINC00862 significantly promoted the proliferation of HCC cells, while its overexpression effectively suppressed this growth *in vitro* (Fig. 2A). Furthermore, we confirmed that LINC00862 markedly inhibited cell growth through colony formation assays (Fig. 2B). The transwell assays also illustrated that LINC00862 could inhibit the migratory and invasive abilities of hepatoma cells (Fig. 2C, D). For *in vivo* experiments, LINC00862 significantly limited HCC growth, impaired subcutaneous tumor development, and suppressed Ki-67 expression (Fig. 3A–E). Additionally, our *in vivo* imaging studies revealed that LINC00862 substantially curtailed the metastatic potential of hepatoma cells (Fig. 3F, G). In summary, our findings confirm that LINC00862 significantly inhibits HCC tumor progression both *in vitro* and *in vivo*.

### RBM47 serves as an indispensable mediator in facilitating the functional roles of LINC00862 in HCC

We overexpressed LINC00862 in HCCLM3 cells and performed RNA sequencing (GEO datasets, GSE234454), which confirmed significant differential expression (6269 up-regulated and 5031 down-regulated) of 11,300 genes (Fig. S2A). Gene Ontology (GO) and Kyoto Encyclopedia of Genes and Genomes (KEGG) analyses on the differentially expressed genes revealed significant enrichments in multiple items and pathways in both analyses (Fig. S2B–E). We then summarized the top 10 entries and pathways with the largest differences and gene enrichment, noting that protein binding and pathways in cancer were the most significant. This suggests that LINC00862 is potentially involved in tumor regulation and may be associated with molecules that exhibit protein-binding functions (Fig. S3A–D). To further pinpoint relevant target pathways, we conducted quantitative proteomics by overexpressing LINC00862 in HCCLM3 cells (iProX datasets, PXD042881), observing significant changes in the expression of 2204 proteins (1039 up-regulated and 1165 down-regulated) (Fig. S4A). Differentially expressed proteins were subjected to GO and KEGG analyses, yielding significant enrichments in various items and pathways (Fig. S4B–D). Notably, we obtained few overlaps between the top 10 entries in GO analysis and RNA sequencing-enriched items, particularly for biological process and molecular function (Fig. S5A, B). This trend persisted in our KEGG analysis (Fig. S5C, D).

**Figure 4:**
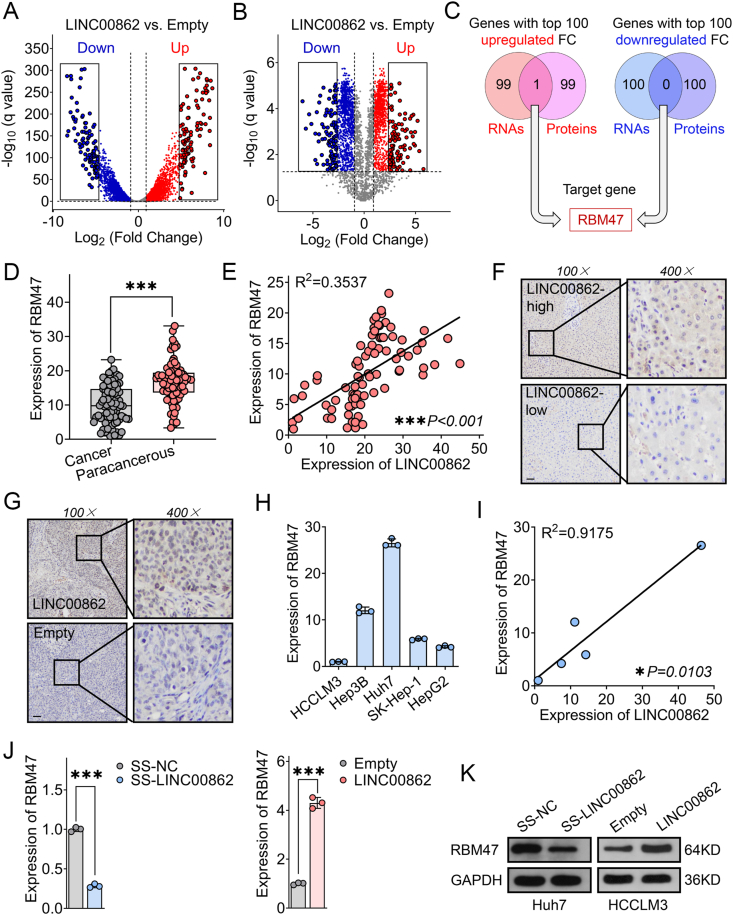
RBM47 serves as a target molecule for LINC00862. **(A, B)** Volcano plots of (A) RNA sequencing and (B) quantitative proteomics data are presented, with the criteria of fold change >2 or <0.5 and Q < 0.05 denoting up-regulation (red) or down-regulation (blue). The top 100 up- or down-regulated genes with respect to fold change are highlighted in a black frame. **(C)** The intersection of the top 100 up- or down-regulated RNAs and proteins was analyzed to identify the target gene. **(D)** The expression of RBM47 was measured by quantitative reverse transcription PCR (qRT-PCR) in both hepatocellular carcinoma (HCC) and paracancerous tissues (*n* = 84 for both). **(E)** A bivariate correlation analysis was performed to assess the relationship between LINC00862 and RBM47 in 84 HCC tissues. **(F)** Immunohistochemistry imaging was used to visualize RBM47 expression in HCC tissues with both high and low expression levels of LINC00862. Black bar = 100 μm. **(G)** Immunohistochemistry imaging was used to visualize the expression of RBM47 in xenografts derived from an empty control group and a LINC00862 overexpression group. Black bar = 100 μm. **(H)** qRT-PCR was employed to assess the levels of RBM47 mRNA in 5 hepatoma cell lines. **(I)** A bivariate correlation analysis was conducted to validate the association between LINC00862 and RBM47 mRNA in 5 hepatoma cell lines. **(J)** qRT-PCR and (K) western blotting were conducted to measure the expression levels of RBM47 in Huh7 cells (in which LINC00862 was silenced) and HCCLM3 cells (in which LINC00862 was overexpressed). ∗*P* < 0.05 and ∗∗∗*P* < 0.001.

**Figure 5:**
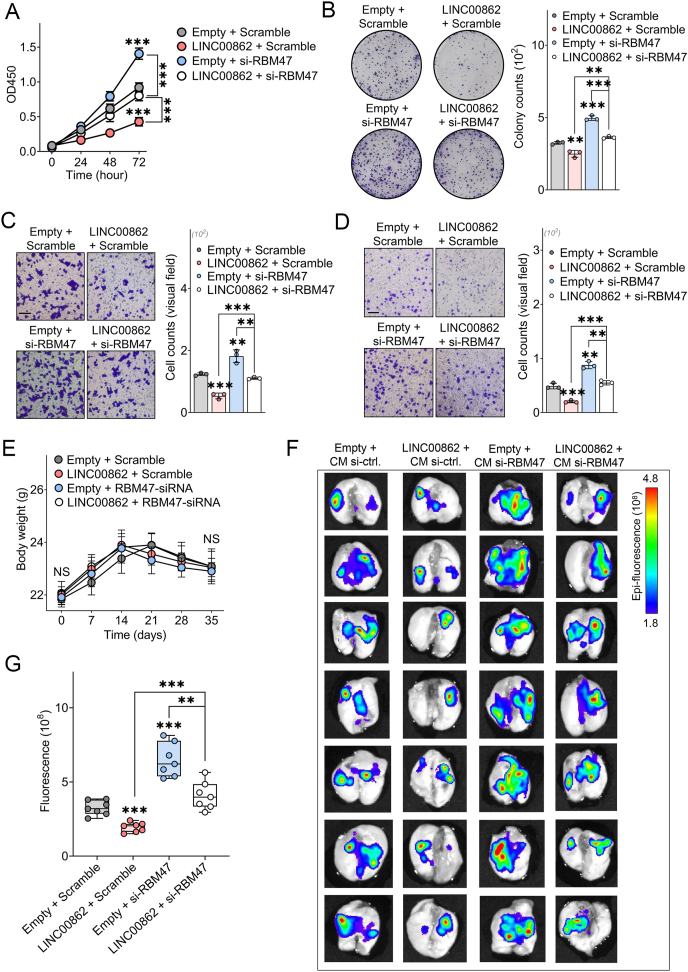
RBM47 delivers the functions of LINC00862 *in vitro* and *in vivo*. **(A)** The proliferation curves of HCCLM3 cells were analyzed after overexpression of LINC00862 and knockdown of RBM47 through the CCK8 assay (*n* = 5). **(B)** The colonizing ability of HCCLM3 cells with LINC00862 overexpression and RBM47 silencing was evaluated through the colony formation assay. **(C, D)** The representative microscopical images of (C) migration and (D) invasion of HCCLM3 cells with LINC00862 overexpression and RBM47 knockdown were analyzed using the transwell assay. Black bar = 100 μm. For the results in (B–D), the experiments were performed in triplicate, and the standard deviation was graphed (*n* = 3). **(E)** Documentation of the weights of nude mice after establishment of the pulmonary metastasis model in each group of rescue experiments (*n* = 7 per group). **(F, G)***In vivo* imaging of lung samples (F) and fluorescence quantitation (G) were analyzed after tail vein injection of HCCLM3 cells that had stably overexpressed LINC00862 and were treated with CM si-RBM47 (*n* = 7 per group). CM, cholesterol-modified. NS, no significance; ∗*P* < 0.05, ∗∗*P* < 0.01, and ∗∗∗*P* < 0.001.

To better identify the primary target genes of LINC00862, we filtered out genes with FPKM (fragments per kilobase of transcript per million fragments mapped) <0.1 and fold change <2 or >0.5 from RNA sequencing, resulting in a set of 5865 differential genes (2194 up-regulated and 3671 down-regulated) (Fig. 4A). Similarly, we excluded genes with invalid comparisons and fold change <2 or >0.5 from quantitative proteomics, revealing significant differential expression of 2131 proteins (1005 up-regulated and 1126 down-regulated) (Fig. 4B). We then identified the top 100 up- or down-regulated genes in both RNA sequencing and quantitative proteomics and performed pairwise intersectional comparison. Our results revealed up-regulation of RBM47, also enriched in the protein binding GO item, whereas no common genes were noted among the top 100 down-regulated genes in RNA sequencing and protein quantification (Fig. 4C). Therefore, we tentatively conclude that RBM47 may be a potential target gene for LINC00862. To further investigate the association between RBM47 and LINC00862, we initially performed qRT-PCR analysis to compare the expression levels of RBM47 mRNA in HCC and paracancerous tissues, which revealed a significant down-regulation in HCC tissues (Fig. 4D). We then went on to assess the correlation between the expression levels of LINC00862 and RBM47 mRNA in 84 HCC tissues and found a robust positive correlation between the two (Fig. 4E). Subsequently, immunohistochemical analyses revealed that RBM47 protein levels were higher in HCC tissues that expressed high levels of LINC00862 (Fig. 4F, G). To validate the correlation between *in vitro* expression of LINC00862 and RBM47, we employed qRT-PCR to assess the expression levels of RBM47 mRNA across 5 hepatoma cell lines (Fig. 4H). Our results demonstrated a consistent positive correlation between LINC00862 and RBM47 mRNA within the hepatoma cell lines (Fig. 4I). Further experiments involving knocking down and overexpressing LINC00862 in Huh7 and HCCLM3 cells, respectively, indicated that RBM47 was a direct target gene of LINC00862 and was positively regulated by it at both the nucleic acid and protein levels (Fig. 4J, K). To demonstrate whether RBM47 played a role in LINC00862-mediated regulation of HCC, we performed rescue experiments in HCCLM3 cells, where RBM47 was knocked down, and LINC00862 was overexpressed. Our results revealed that RBM47 knockdown abrogated the effects of LINC00862 on cell proliferation and growth (Fig. 5A, B), while the inhibitory effect of LINC00862 on the inhibition of cell migration and invasion was substantially diminished when RBM47 was silenced (Fig. 5C, D). Additionally, using cholesterol-modified siRNA to interfere with RBM47 weakened LINC00862's ability to inhibit lung metastasis *in vivo* (Fig. 5E–G). Thus far, these results demonstrate that RBM47 is a direct target gene of LINC00862 and its expression levels modulate LINC00862-mediated anti-cancer effects.

### The promoter of RBM47 represents a direct target of LINC00862 through triplex formation

**Figure 6:**
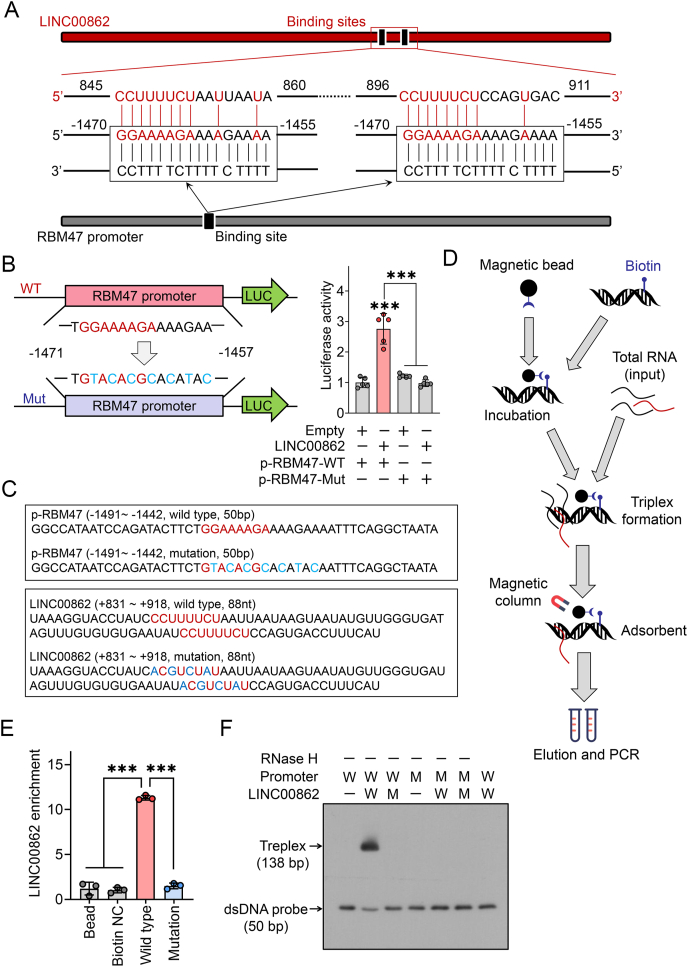
LINC00862 binds to the RBM47 promoter by forming a triplex. **(A)** The binding sites between LINC00862 and RBM47 promoter are presented, based on the pyrimidine motif of Hoogsteen base pairing. **(B)** Promoter activity of RBM47-WT and RBM47-Mut was quantitatively analyzed after LINC00862 transfection using the dual luciferase reporter system. **(C)** The dsDNA probe sequences of RBM47-WT promoter and RBM47-Mut promoter (upper) and the RNA probe sequences of LINC00862-WT and LINC00862-Mut (lower) are presented. The red-marked sequence denotes the region of molecular interaction where binding occurs. The blue-labeled nucleotide denotes the location of a genetic mutation. **(D)** The triplex pulldown assay is elucidated schematically. Total RNAs were pulled down by biotin-labeled dsDNA probe for quantitative reverse transcription PCR validation. **(E)** LINC00862 was quantitatively analyzed after being pulled down by RBM47 promoter probes using quantitative reverse transcription PCR. **(F)** Triplex formation of LINC00862 and RBM47 promoter was detected through electrophoretic mobility shift assay. RNase H was applied to eliminate any DNA-RNA triplexes for the negative control. WT and W, wild type; Mut and M, mutant type. ∗∗∗*P* < 0.001.

Intranuclear lncRNAs have been shown to have diverse roles in transcriptional regulation through either cis or trans interactions, activating or inhibiting downstream transcription of genes.^8^^,^^23^ Within the promoter region of RBM47, only one homopurine stretch (−1470/−1455) has been identified and may directly bind to LINC00862 through Hoogsteen pairing to regulate transcription. Two potential binding sites (+845/+859 and + 896/+908) for LINC00862 were identified through sequence alignment with NCBI Nucleotide (Fig. 6A). To confirm this binding interaction, wild-type and putative binding site-mutated RBM47 promoter probes were constructed and transfected with LINC00862 into HCCLM3 cells using a dual luciferase reporter system to evaluate promoter activity. The results indicated that the putative binding site significantly influenced the activity of the RBM47 promoter, suggesting that LINC00862 may promote RBM47 transcription through this site (Fig. 6B). To directly demonstrate the binding of LINC00862 with the RBM47 promoter, wild-type and mutated double-stranded DNA (dsDNA) probes of the homopurine stretches region were constructed and validated for binding through a triplex pulldown assay (Fig. 6C). qRT-PCR analysis demonstrated a notable enrichment of LINC00862 in pulled-down RNA from the wild-type dsDNA probe in comparison to that of the mutated probe (Fig. 6D, E). Furthermore, we synthesized the RNA probe that aligns with the sequence of +831/+918 on LINC00862 (Fig. 6C), encompassing the binding region. The electrophoretic mobility shift assays showed that in the presence of wild-type LINC00862 RNA probe containing the binding region, the wild-type dsDNA probe can form a triplex structure, while RNase H served as a negative control (Fig. 6F). These findings strongly suggest that LINC00862 binds to the RBM47 promoter region through Hoogsteen pairing, leading to the promotion of RBM47 transcription.

### CHD5 is implicated in the regulation of LINC00862 for RBM47

**Figure 7:**
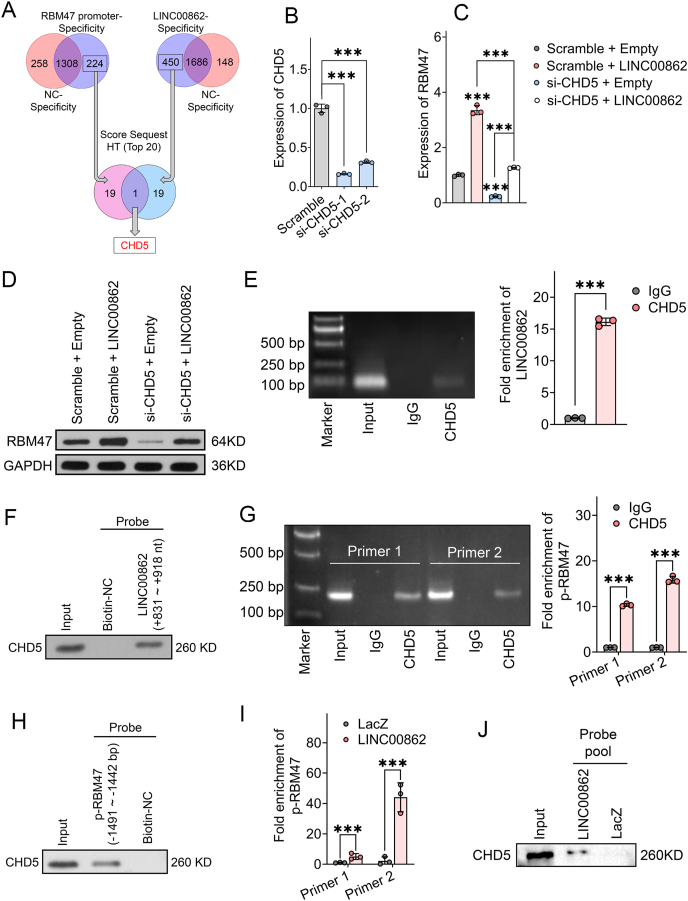
CHD5 participates in the transcriptional regulation of LINC00862 on RBM47. **(A)** A Venn diagram is used to elucidate the screening process of target proteins that co-interact with the RBM47 promoter and LINC00862. **(B)** Following the transfection of siRNAs in HCCLM3 cells, quantitative reverse transcription PCR (qRT-PCR) was employed to assess the knockdown efficiency of CHD5. The si-CHD5-1 was chosen for subsequent experimental investigations. **(C, D)** The expression of RBM47 is detected through (C) qRT-PCR and (D) western blotting after CHD5 silencing and LINC00862 overexpression. **(E)** Electrophoresis and qRT-PCR were utilized to evaluate the enrichment of LINC00862 normalized to IgG. **(F)** The CHD5 band was detected through western blotting after the RNA pulldown of the LINC00862 probe. **(G)** Electrophoresis and qRT-PCR were used to evaluate the enrichment of the RBM47 promoter after chromatin immunoprecipitation. **(H)** Western blotting was performed to detect CHD5 pulled by the RBM47 promoter via DNA pulldown assay. **(I, J)** qRT-PCR (I) and western blotting (J) were utilized to detect the enrichment of RBM47 promoter and CHD5, respectively, after chromatin isolation by RNA purification. NC, negative control. ∗*P* < 0.05, ∗∗*P* < 0.01, and ∗∗∗*P* < 0.001.

Our experimental data conclusively confirm the functional relevance of the LINC00862-RBM47 promoter binding relationship in modulating transcriptional activity in the latter. Accordingly, we postulated that this phenomenon might be facilitated by certain proteins or transcription factors that form transcriptional complexes to maximize RBM47 transcriptional output. The hypothesis was then probed through pulldown assays using DNA and RNA probes (Fig. 6C; Table S2), and the implicated proteins were subjected to mass spectrometry analysis. Our results showed that among the 1532 genes enriched by the RBM47 promoter probe, 224 gene products were specifically bound to the probe, as compared with a biotin control (iProX datasets, PXD042883). Furthermore, the proteins bound by the LINC00862 probe were found to be 2136 in total, with 450 showing a specific association (Fig. 7A) (iProX datasets, PXD042884). Notably, GO and KEGG analyses of both sets of proteins indicated significant enrichment in various biological pathways. Although we observed some overlaps in certain pathways, such as endocytosis, it was evident that tumor-related and transcriptional regulation-related pathways had disparate associations with the DNA and RNA probes (Fig. S6A–D; Fig. S7A–D).

After the mass spectrometry analysis of the DNA and RNA pulldowns, we proceeded to orthogonalize the Sequest HT scores of the top 20 proteins according to the obtained results (Table S3, 4). With this method, we identified CHD5 as a putative gene product involved in this cross-talk (Fig. 7A). To validate the correlation between CHD5 and the LINC00862/RBM47 axis, we assessed the levels of CHD5 protein in tissue samples from HCC patients categorized into the high LINC00862/RBM47 and low LINC00862/RBM47 groups. The results revealed a significant up-regulation of CHD5 protein levels in HCC tissues characterized by high expression of LINC00862 and RBM47 (Fig. S8A–C). Overall, there was a pronounced positive correlation between CHD5 protein levels and the RNA expression of LINC00862 and RBM47 in HCC tissues (Fig. S8D, E). This positive correlation was also observed in HCC cell lines (Fig. S8F–H). In further exploring the regulatory role of CHD5 on RBM47, knockdown of CHD5 was carried out in the HCCLM3 cell line (Fig. 7B). We discovered that CHD5 could up-regulate the expression of both RBM47 mRNA and protein, and this effect required the involvement of LINC00862 (Fig. 7C, D). These results suggested that CHD5 may serve as an upstream regulator influencing RBM47 mRNA expression. To further elucidate whether CHD5's regulation of the LINC00862/RBM47 axis directly impacted oncogenic phenotypes, we knocked down CHD5 in HCCLM3 cells while simultaneously overexpressing LINC00862 and RBM47. The results from functional experiments indicated that CHD5 could suppress tumor behaviors, and upon knockdown of CHD5, the tumor suppressive effects of LINC00862 and RBM47 were significantly diminished. This suggests that interfering with CHD5 could block oncogenic phenotypes driven by the LINC00862-RBM47 axis (Fig. S9A–D; Fig. S10A–D). Mechanistically, given CHD5's DNA-binding characteristics, we speculated that LINC00862 may recruit CHD5, thereby forming a multimeric complex that positively induces the transcription of RBM47. To validate our assumption, we employed RNA immunoprecipitation-PCR and RNA pulldown assays that confirmed direct binding between CHD5 and LINC00862 (Fig. 7E, F). The results of chromatin immunoprecipitation-PCR and DNA pulldown assays were in concurrence with these findings, indicating direct binding of CHD5 to the RBM47 promoter (Fig. 7G, H). Finally, we used chromatin isolation by RNA purification-PCR and chromatin isolation by RNA purification-western blotting techniques, which confirmed direct interaction among all three components (Fig. 7I, J; Table S2), thus substantiating our hypothesis that CHD5 acts as a transcription factor recruited by LINC00862 to form a transcriptional complex that maximizes RBM47 transcription.

To further ascertain the specific binding region of CHD5 on LINC00862, we engineered a LINC00862 vector with a precise deletion of the nucleotide region spanning from +831 to +918 (+831/+918) (LINC00862-De vector). We then generated RNA probes specifically designed for the full-length sequence of the LINC00862 transcript (LINC00862-Fu probe) as well as the LINC00862 deletion sequence (LINC00862-De probe) corresponding to the LINC00862 vector and LINC00862-De vector, respectively. We subsequently performed RNA pulldown experiments, which revealed that CHD5 did not exhibit interaction with LINC00862-De probe (Fig. S11A). Chromatin immunoprecipitation-PCR analysis also revealed that the enrichment of CHD5 at the RBM47 promoter mediated by LINC00862-De transfection was significantly lower than that observed with the transfection of the wild-type LINC00862 (Fig. S11B). These observations indicate that the +831/+918 region of LINC00862 functions as the specific binding region for CHD5. Furthermore, in contrast to the overexpression of full-length LINC00862, the up-regulation of LINC00862-De appeared to have no significant impact on the oncogenic phenotypes observed in both *in vitro* and *in vivo* models (Fig. S11C–F; Fig. S12A–F). These findings highlight the significance of the +831/+918 region as the specific binding region for CHD5 on LINC00862, as well as its essential role as the core region responsible for the inhibitory function of LINC00862 in HCC.

### RBM47 exerts a positive regulatory influence on LINC00862 as a transcription factor

**Figure 8:**
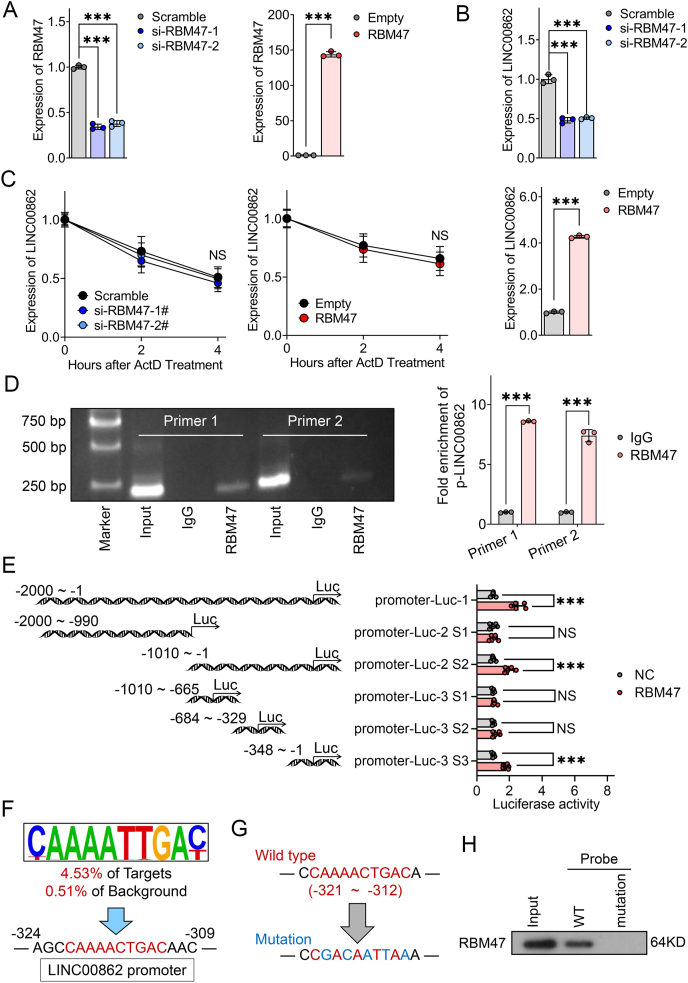
RBM47 acts as a transcription factor of LINC00862. **(A**–**C)** After silencing RBM47 in Huh7 cells and overexpressing RBM47 in HCCLM3 cells, quantitative reverse transcription PCR was used to detect the expression of (A) RBM47 and (B) LINC00862, and (C) an RNA decay assay was performed to assess the degradation of LINC00862. **(D)** Electrophoresis and quantitative reverse transcription PCR were used to detect the enrichment of LINC00862 promoter after chromatin immunoprecipitation. **(E)** The dual luciferase reporter system tested the promoter activity of LINC00862 after RBM47 transfection. Truncated regions of the promoter and corresponding quantitative analyses are presented from top to bottom. **(F)** The putative binding motif of RBM47 and the corresponding binding site on LINC00862 promoter are presented. **(G)** The wild-type and mutant DNA probes of the LINC00862 promoter containing the putative binding site recognized by RBM47 are presented. **(H)** Western blotting was used to test the RBM47 pulled by the LINC00862 promoter through DNA pulldown assay. ActD, actinomycin D. NS, not significant; ∗∗∗*P* < 0.001.

**Figure 9:**
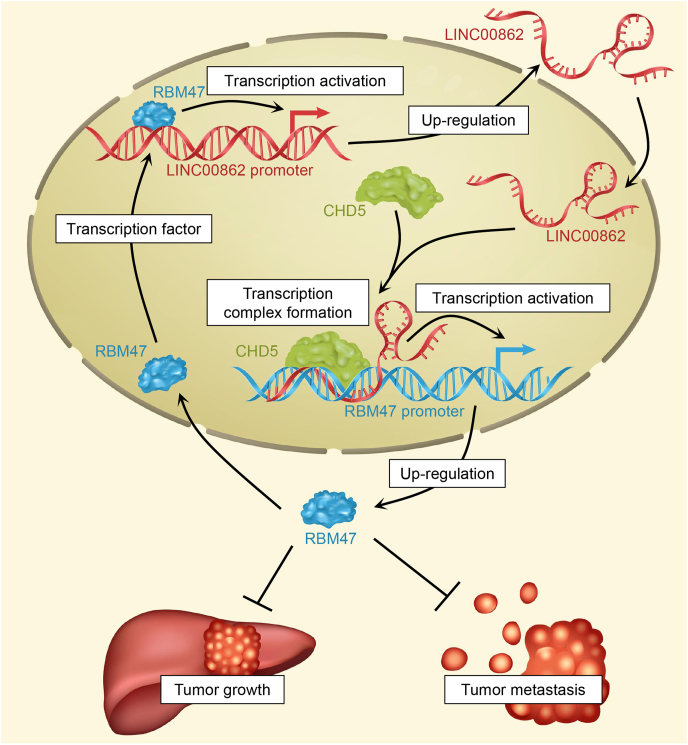
Schematic representation of the molecular mechanism underlying LINC00862-mediated suppression of hepatocellular carcinoma progression via RBM47. LINC00862 forms a triplex with the RBM47 promoter region within the nucleus, and in doing so, recruits CHD5 to facilitate the up-regulation of RBM47 transcription and expression. Furthermore, RBM47 can directly function as a transcription factor to facilitate the transcription of LINC00862. Consequently, LINC00862 and RBM47 mutually positively regulate each other via positive feedback, instigating the inhibition of hepatocellular carcinoma progression.

Following our identification of the positive regulatory effect of LINC00862 on RBM47, we set out to explore whether RBM47 reciprocally regulated LINC00862. To this end, we performed experiments to knock down RBM47 in Huh7 cells and overexpress it in HCCLM3 cells (Fig. 8A), followed by qRT-PCR analysis to quantify LINC00862 expression levels. Our data revealed a significant up-regulation of LINC00862 by RBM47 in hepatoma cells (Fig. 8B). As an RBP, RBM47 has been demonstrated to possess direct RNA-binding capability and exert regulatory effects on RNA stability.^13^ We achieved RBM47 knockdown in Huh7 cells and undertook RNA decay experiments to assess the alterations in LINC00862 degradation. The outcomes unveiled insignificant modifications in the stability of LINC00862 after RBM47 knockdown. Meanwhile, we proceeded to overexpress LINC00862 in HCCLM3 cells and likewise observed no conspicuous shifts in the stability of LINC00862 (Fig. 8C). Hence, we deduce that RBM47 does not exert a pronounced influence on the stability of LINC00862. Based on these results, we hypothesize that RBM47 might modulate the transcriptional level of LINC00862. Through chromatin immunoprecipitation-PCR assays performed on the LINC00862 promoter, we demonstrated a marked enrichment of RBM47 protein (Fig. 8D). The dual-luciferase reporter system confirmed that RBM47 could promote the transcription of LINC00862. Moreover, we observed that RBM47 protein primarily activated LINC00862 transcription within the −348/−1 promoter region (Fig. 8E). Leveraging previous Homer *de novo* motif dataset (based on chromatin immunoprecipitation sequencing, GEO datasets, and GSE201818) and the MEME Suite online prediction tool,^24^ we identified an RBM47 binding motif within the truncated target region (*P* = 9.08 × 10^−6^, *Q* = 0.0167), providing insights into the mechanism of RBM47 protein-LINC00862 promoter interaction (Fig. 8F). Finally, through DNA pulldown assays with both wild-type and mutated probes of the target region, we confirmed the direct association of RBM47 protein to the LINC00862 promoter (Fig. 8G, H). To summarize, our research has elucidated the molecular mechanisms underlying the transcriptional activation of LINC00862 by RBM47, leading to a concomitant up-regulation. Of note, we have uncovered a bidirectional regulatory relationship between LINC00862 and RBM47, which mediates the suppression of HCC growth and metastasis (Fig. 9).

## Discussion

LINC00862 is a novel lncRNA with a single transcript of 1445 nucleotides transcribed from chromosome 1. Liu et al have found that LINC00862 is up-regulated in most tumors and can be used as a biomarker to predict the efficacy of immunotherapy. At the same time, it can regulate the expression of SIRT1 in cervical cancer and gastric cancer through the ceRNA mechanism.^25^ On the contrary, our extensive clinical sample analysis and characteristics have demonstrated the differential expression and prognostic impacts of LINC00862 in HCC with extraordinary clinical significance. Our investigations have further established a direct correlation between LINC00862 expression levels and tumor pathology grading as well as tumor diameter, implying its promising potential as a valuable biomarker for HCC. In addition, our results have astutely confirmed the primary localization of LINC00862 within the confines of the cell nucleus. As previously denoted, nuclear-localized lncRNAs have the adept ability of mediating gene expression through transcriptional regulation, which operates as one of the fundamental mechanisms by which they maintain and control target genes.^7^^,^^26^^,^^27^ Wang et al and Yang et al previously reported on the intricate regulation of transcriptional initiation via lncRNA HEGBC and DILC's interaction with the single-stranded DNA promoter region of target genes through Watson-Crick pairing.^28^^,^^29^ However, the widely acknowledged triple-helix formation based on the Hoogsteen pairing principle is prominent in the binding of DNA and RNA. lncRNAs can form parallel or anti-parallel orientation with purine-rich sites on the double-stranded DNA via Hoogsteen pairing, providing a mechanism for binding to DNA, as demonstrated in the interaction between lncRNA and DNA.^30^ Kalwa et al have previously reported HOTAIR's ability to regulate transcriptional regulation by binding to the double-stranded DNA promoter sequence of target genes in an anti-parallel orientation through Hoogsteen pairing with its own purine motif, thereby promoting notable changes in mesenchymal hepatocyte differentiation.^31^ In tumorigenesis, Postepska et al have uncovered that lncRNA Khps1 achieves complex control of cell apoptosis through the formation of a triple-helix structure with the target gene promoter region, based on pyrimidine motifs in a parallel orientation via Hoogsteen pairing,^32^ which also aligns with the prior study by Fang et al for lung adenocarcinoma.^33^ Our research has likewise unveiled the parallel orientation-based binding of LINC00862 to the RBM47 promoter region via pyrimidine motifs interacting with purine-rich stretches, consistent with previous reports.

Despite the existence of direct transcriptional regulation functions for lncRNAs, our research posits that LINC00862 primarily acts as a molecular guide factor, responsible for facilitating the targeting and regulation of target gene transcription by functional proteins. Specifically, our study reveals that CHD5 is a regulatory factor that interacts with both LINC00862 and the RBM47 promoter, and plays a significant role in LINC00862-mediated transcriptional regulation of RBM47. The region on LINC00862 that pairs with the RBM47 promoter is also a specific binding region for CHD5. This region exerts a direct influence on the association between CHD5 and the RBM47 promoter, and concurrently serves as a pivotal region crucial for LINC00862-mediated inhibition of HCC functionality. Therefore, the involvement of both LINC00862 and CHD5 is indispensable in the transcriptional modulation of the RBM47 promoter, and their regulatory actions upon the RBM47 promoter are mutually dependent. As a chromatin remodeling factor, CHD5's transcriptional regulation abilities have been established in multiple studies.^14^^,^^34^ Furthermore, CHD5 has been identified as a tumor suppressor that exerts inhibitory roles across a range of cancer types.^18^ In the context of HCC, both Zhao et al and Xie et al have demonstrated significant down-regulation of CHD5, further underscoring its critical influence on HCC's biological behavior.^35^^,^^36^ Our study demonstrates a positive correlation between CHD5 and the levels of LINC00862/RBM47. Furthermore, CHD5 exerts its influence on oncogenic phenotypes in HCC through the LINC00862-RBM47 axis. These findings provide a comprehensive understanding of CHD5's role in HCC from multiple perspectives and further substantiate and reinforce previous investigations. On the other hand, given the screening threshold for interactive proteins, it is imperative to acknowledge the possibility that CHD5 may not be the exclusive regulating factor implicated in the regulatory process. Moreover, the findings from mass spectrometry analysis suggest the potential involvement of multiple RNA-binding proteins that could potentially interact with DNA (Table S3). In other words, it is conceivable that several known RNA-binding proteins potentially possess latent DNA-binding capabilities.^13^^,^^37^ These observations highlight the prospects for future investigations to delve deeper into this domain and uncover novel regulatory factors associated with this process.

We substantiate that RBM47 is positively regulated by LINC00862, serving as its target molecule. However, the role of RBM47 in tumorigenesis remains a subject of debate.^38^ Shen et al and Sakurai et al have respectively shown that RBM47 restricts the progression of non-small cell lung cancer and lung adenocarcinoma.^39^^,^^40^ Additionally, Rokavec et al and Vanharanta et al have respectively investigated that RBM47 functions as a tumor suppressor in colon and breast cancer.^41^^,^^42^ On the contrary, in nasopharyngeal carcinoma, Xu et al have found that RBM47 promotes tumorigenesis.^43^ Nevertheless, our previous research has shown that RBM47 significantly inhibits HCC progression,^13^ as consistent with some prior studies. Mechanistically, as an RNA-binding protein, RBM47 may predominantly regulate alternative RNA splicing.^11^^,^^44^ However, at the transcriptional level, RBM47 protein has demonstrated its function as a transcription factor by directly binding to promoter regions and regulating transcription.^12^^,^^13^^,^^43^ Our study has initially demonstrated that RBM47 exerts negligible influence on the stability of LINC00862, thus indicating that the regulation of LINC00862 may predominantly transpire at the transcriptional level. Meanwhile, we confirmed RBM47's ability to serve as a transcription factor for LINC00862, which subsequently activates a positive feedback loop where RBM47 up-regulates itself while simultaneously counter-regulating LINC00862's function. This feedback loop exemplifies a complex and dynamic mechanism of regulatory control in transcriptional processes.

This study represents a groundbreaking discovery, as it sheds light on the clinical expression disparity of functional LINC00862 and its prognostic function. We have also provided unprecedented insight into the molecular functions of LINC00862 in HCC, substantiating its anti-cancer activity. Moreover, our research has revealed the interplay between lncRNAs and RNA-binding proteins in a positive feedback loop, which may signify the existence of multiple mechanisms involved in tumor regulation. Additionally, we have confirmed the transcriptional regulatory roles of CHD5 and RBM47. On the other hand, we must humbly recognize the limitations of our study. Firstly, whilst we have established the clinical characteristics of LINC00862, additional validation of its clinical relevance is warranted, given the present constraints on sample size. What's more, while we have observed two potential binding sites for the RBM47 promoter in close proximity on LINC00862, their respective effectiveness has yet to be explored thoroughly. Furthermore, despite the established contribution of CHD5 to RBM47 regulation, extensive research into the underlying regulatory mechanisms is yet to be conducted. Previous research has suggested that CHD5 predominantly participates in transcriptional regulation via histone methylation maintenance.^14^^,^^34^ However, our study postulates that the mechanism by which CHD5 positively regulates RBM47 is potentially associated with histone acetylation,^45^ a fascinating research direction that holds much promise.

In summary, our results cumulatively demonstrate the pivotal role of RBM47 as a transcription factor that drives the up-regulation of LINC00862. Moreover, LINC00862 forms a complex in synergy with CHD5, which binds to the RBM47 promoter, thereby enhancing RBM47 transcriptional activity and contributing significantly to the suppression of HCC progression. Consequently, we present compelling evidence supporting the presence of a positive feedback loop between RBM47 and LINC00862, which has a significant impact on the inhibition of HCC tumorigenesis.

## CRediT authorship contribution statement

**Tao Guo:** Project administration, Conceptualization, Writing – original draft, Data curation, Funding acquisition. **Yingying Jiang:** Software, Resources, Data curation, Writing – original draft, Formal analysis, Funding acquisition. **Shunshun Zhu:** Investigation, Software, Visualization, Funding acquisition, Methodology. **Min Shi:** Methodology, Software, Investigation. **Linying Sun:** Software, Methodology, Validation, Resources, Investigation. **Juan Feng:** Visualization, Software, Resources. **Zhen Li:** Visualization, Supervision, Conceptualization, Validation, Writing – review & editing, Project administration. **Cheng Gong:** Writing – review & editing, Project administration, Supervision, Funding acquisition, Visualization.

## Ethics declaration

Anonymous clinical sample collection and data analysis were authorized by the Human Subjects Committee of Zhongnan Hospital of Wuhan University (Approval Number: 2021108), and all included patients provided signed informed consent. Animal studies were approved by the Ethics Committee of Weifang Medical University approved the related protocols (Approval Number: 2021SDL248).

## Data availability statement

The authors declare that data supporting the findings of this study are available upon reasonable request. The complete RNA-seq data were uploaded to the Gene Expression Omnibus (GSE234454, https://www.ncbi.nlm.nih.gov/geo/). Data of proteomics and mass spectrometry was uploaded to the Integrated Proteome Resources (PXD042881, PXD042883, PXD042884, https://www.iprox.cn/page/home.html).

## Funding

This work was by the National Natural Science Foundation of China (No. 82103166), Natural Science Foundation of Shandong Province, China (No. ZR2021QH200, ZR2020MH192), Youth Innovation Technology Project of Higher School in Shandong Province, China (No. 2022KJ267), Research Start-up Funds of Shandong Second Medical University, Shandong, China (No. 04102001, 02194001), and Science and Technology Innovation Cultivation Fund of Zhongnan Hospital of Wuhan University, Wuhan, China (No. znpy201802).

## Conflict of interests

The authors declared no competing interests.
