# Supplementary material for: The role of positive feedback loop between LINC00862 and RBM47 in hepatocellular carcinoma suppression
Source: Genes Dis. 2025 Jul 9;13(2):101763. doi: 10.1016/j.gendis.2025.101763 (PMC12757521; doi:10.1016/j.gendis.2025.101763)
Supplement: Multimedia component 1 [file mmc1.pdf]

Supplementary methods

Quantitative Reverse Transcription-PCR (qRT-PCR)

RNA extraction was conducted utilizing the RNA Rapid Extraction Kit (Fastagen, 220011) in adherence to the established protocols. Total RNA quantity was evaluated with the NanoDrop2000 (Implen, N50) instrument, with samples exhibiting an A260/A280 ≥ 1.9 being utilized in downstream analyses. The ReverTra Ace qPCR RT Kit (Toyobo, FSQ-101) was utilized for reverse transcription (RT) for a duration of 15 minutes at 37℃. Subsequently, quantitative PCR was conducted over 40 cycles utilizing the Prism® 7900HT (ABI, 7900HT) instrument, with SYBR® Premix (Toyobo, QPK-212), primers (Supplementary Table S1), and ddH_2O included in a 20μL reaction system. To control the variance introduced during sample processing, GAPDH and U6 were utilized as internal reference controls for normalization of total RNA and intranuclear RNA, respectively. Finally, quantitative comparisons were obtained utilizing the $2^{-\Delta\Delta Ct}$ calculation.

Western blotting

RIPA lysis buffer (Beyotime, P0013B) was utilized for the extraction of cell and tissue proteins prior to separation through gel electrophoresis using the PAGE Gel Fast Preparation Kit (Epizyme Biotech, PG110, and PG112) at 120V. To achieve transmembrane protein migration, a PVDF membrane (Millipore, IPVH00010) was employed for a duration of between 30 minutes to 2 hours based on the molecular weight at a constant current of 300mA. Following transfer, a mixture of either anti-RBM47 (Proteintech, 23902-1-AP, 1:2000) or anti-CHD5 (Cell Signaling Technology, #44829, 1:1000), and Skim Milk (Solarbio, D8340), was utilized for overnight incubation at 4℃. Protein band development was accomplished via the utilization of the highly sensitive Affinity® ECL Kit (Femtogram, KF8003), with commercial marker (Epizyme, WJ102) oriented towards scan purposes. Quantitative analysis of protein blot intensities was performed using Image Pro Plus software (version 6.0).

RNA fluorescent in situ hybridization (FISH)

RNA Fluorescence In Situ Hybridization (RNA FISH) analysis was performed using the FISH Probe Kit (RiboBio, C10910) in adherence to the manufacturer's guidelines. Following fixation with 4% polyformaldehyde, Huh7 and HCCLM3 cells were incubated with pre-cooled 5% Triton™ X-100 (Sinopharm, 30188928) for a brief 5-minute period at a temperature of 4℃. Subsequently, hybridization buffer and the LINC00862 probe (produced by RiboBio) were allowed to incubate overnight at 37℃ under complete darkness. After hybridization completed, nuclei were stained using DAPI (Solarbio, S2110) with U6 and 18S hybridized as subcellular reference controls. Finally, cellular visualization was accomplished through confocal microscopy (Perkin Elmer & Olympus, UltraVIEW VoX & IX81) after sealing with nail polish.

Cell proliferation

Subsequent to transfecting hepatoma cells, cell proliferation was assessed using the Cell Counting Kit-8 (CCK-8) (Solarbio, CA1210). A total of 1×10^3 cells were seeded in individual wells of a 96-well plate containing 100μL of complete medium, and then incubated at 37℃ for 72 hours. Afterward, 10μL of the CCK-8 solution was added to the medium and allowed to incubate for 2 hours to detect cell proliferation. Finally, absorbance readings were taken at a wavelength of 450 nm using a microplate reader (Kehua Bio-Engineering, ST-360).

Colony formation

Following trypsin treatment, transected hepatoma cells were resuspended in complete medium to allow for individual cell identification. The cells were then seated in a 6-well plate and incubated at 37℃ for 2 weeks. Post-incubation, the cells were washed with PBS buffer and fixed using 4% polyformaldehyde (Sinopharm, 80096618) for a duration of 15 minutes. Subsequently, the cells underwent staining following the application of Crystal Violet Dye Solution (Beyotime, C0121) for 20 minutes, enabling the counting of colonies to be carried out.

Transwell assay

To evaluate cellular migration, transfected cells were resuspended in serum-free DMEM. Subsequently, 1×10^4 cells were seeded in the upper chamber of a 24-well

plate, with DMEM medium containing 10% FBS added to the communicating lower well. Prior to cellular invasion, the bottom of the chamber was coated using Matrigal (BD Biocoat, 356234) with a DMEM dilution range of 1:8. After incubating the hepatoma cells for a duration of 24 hours, they were fixed with 4% polyformaldehyde and stained with Crystal Violet Dye Solution for a period of 20 minutes. The cells were then microscopically photographed for further evaluation.

In vivo experiments

Male 6-8 week-old BALB/c nude mice (Gembio Biotech) were utilized for the *in vivo* experiments of tumor growth and metastasis. 5×10^6 HCCLM3 cells stably transfected with LINC00862/empty vector with fluorescence labeling were subcutaneously injected into the armpits (7 mice in each group) for the evaluation of tumor growth, while 1.5×10^6 cells were injected into the tail vein (7 mice per group) for tumor metastasis observation. Subsequent to a 5-week subcutaneous injection period, mice were sacrificed and tumor growth curves were generated. Meanwhile, subcutaneous tumors were utilized for further staining. For the metastatic tumor model, the lungs of mice in each group were extracted from the pleural cavity for *in vivo* imaging after a 5-week tail vein injection period. To elucidate the function of the CHD5-binding sequence within LINC00862, we developed HCCLM3 cell lines that stably express the LINC00862 deletion sequence (LINC00862-De) construct, which is tagged with the aforementioned fluorescent label, for in vivo growth and metastasis experiments. In the rescue experiments, 15 nmol cholesterol-modified RBM47 siRNA or control siRNA (RiboBio) was injected into the tail vein every 5 days for a duration of 30 days. Finally, tumor metastasis was assessed based on the detection of lung fluorescence intensities utilizing an IVIS® Lumina III System (PerkinElmer).

Hematoxylin and eosin (HE) and Immunohistochemistry (IHC) staining

Following 3 rounds of paraffin (Sinopharm, 69019361) embedding at 60°C, the tissue was sliced and baked at the same temperature for a duration of 3 hours. Slice dewaxing was accomplished through utilization of dimethylbenzene (Sinopharm, 10023418) and ethanol (Sinopharm, 10009218). Hematoxylin (Sigma-Aldrich, H9627) was utilized to stain the slices for a duration of 5 minutes, after which 1%

water-soluble eosin (Sinopharm, 71014544) dye was utilized to stain for the same duration. For IHC staining, slices were immersed and boiled with 0.01M EDTA, pH 9.0 for 15 minutes, following which they were incubated with 3% hydrogen peroxide for 15 minutes at room temperature. Subsequently, anti-Ki-67 (Bioss, bsm-60738R, 1:200), anti-RBM47 (Abcam, ab167164, 1:200), anti-CHD5 (Cell Signaling Technology, #44829, 1:200) and DAB Color Reagent Kit (Servicebio, G1212-200T) were utilized for semiquantitative microscopic evaluation.

RNA decay assay

We conducted an RNA decay assay utilizing actinomycin D (ActD) to assess the stability of RNA. Hepatoma cells were collected from 6-well plates at 0, 2, and 4 hours with the treatment of 5 μg/mL of ActD. Subsequently, the relative expression levels of LINC00862 were analyzed through qRT-PCR, with the RNA levels at 0 hours serving as the baseline reference and being normalized to a value of 1.

RNA sequencing

RNA extraction was carried out after LINC00862 overexpression in HCCLM3 cells utilizing Trizol reagent (ThermoFisher, 15596018). Following extraction, RNAs were selectively captured with Dynabeads™ Oligo(dT)$_{25}$ (ThermoFisher, 25-61005) and subsequently fragmented with Magnesium RNA Fragmentation Module (NEB, E6150S) under 94℃ for 5 minutes. Reverse transcriptase from SuperScript^{TM} II Reverse Transcriptase (Invitrogen, 1896649) was deployed to generate cDNA. U-labeled second-stranded DNAs were synthesized by incubation with DNA polymerase I (NEB, m0209), RNase H (NEB, m0297), and dUTP Solution (ThermoFisher, R0133). Each blunt end was appended with an A base to connect to the indexed adapter which contained a T base. The second-stranded DNAs were subsequently treated with UDG enzyme (NEB, m0280) to prepare for amplification with PCR [1]. The PCR condition comprised initial denaturation for 3 minutes at 95℃, denaturation for 15 seconds at 98℃, annealing for 15 seconds at 60℃ with 8 cycles, extension for 30 seconds at 72℃, ended by a 5-minute extension at 72℃. Following cDNA library construction, RNA sequencing was undertaken on an illumina Novaseq^{TM} 6000 (LC Bio Technology) with the model of PE150. Reads and

Fragments Per Kilobase per Million (FPKM) aligning with each gene were analyzed at the levels of enrichment. The raw data would be directly uploaded to the GEO database [2]. For analyzing, a q value was computed to present differences between groups, and <0.05 was deemed significant. Gene Ontology (GO) and Kyoto Encyclopedia of Genes and Genomes (KEGG) enrichment were analyzed for pathway addressing, and $q < 0.05$ was deemed significantly enriched. Prior to determining target genes, any genes with FPKM < 0.1, fold change (FC) < 2 or > 0.5, or $q > 0.05$ were excluded. The top 100 upregulated and 100 downregulated genes with the most significant FC after LINC00862 overexpression were chosen as potential target genes.

Label-free quantitative proteomics

Label-free quantitative proteomics was conducted subsequent to LINC00862 overexpression in HCCLM3 cells. Following a one-step process of denaturation, reduction, and alkylation at 60°C for a duration of 1 hour, the protein samples were incubated with Trypsin (50:1) at 37°C and shaken overnight for enzyme digestion. Mass spectrometry (MS) data were collected utilizing the Q Exactive™ HF Mass Spectrometer in series with the UltiMate™ 3000 RSLC nano liquid chromatography-mass spectrometry system following termination and desalination of the enzyme digestion. The peptide sample was subsequently separated utilizing a C18 analytical column (75μm x 25cm) (Aurora Ultimate). A 60-minute analysis gradient was established utilizing 2 mobile phases (Phase A: 0.1% formic acid, 3% DMSO; Phase B: 0.1% formic acid, 3% DMSO, and 80% acetonitrile). The flow rate of the liquid phase was set to 300 nL/min with a Data-Dependent Acquisition (DDA) model for data collection. MS data were retrieved utilizing MaxQuant software (V1.6.6) utilizing the Andromeda algorithm based on the Uniprot database [3]. Label-free quantification (LFQ) intensity was utilized for protein quantification analysis [4]. In cases where a sample displays missing detection values, imputation techniques will be used based on the nature of the missing values [5]. However, if two or more samples from each group exhibit missing values or if all values in any group are missing, comparisons of that group will be considered invalid. The unprocessed data shall be retrieved through our online submission on the iProX database [6-7]. Similar to RNA

sequencing, $q < 0.05$ was recognized as significant FC, and the top 100 aberrant proteins were identified as potential target genes of LINC00862. For the analysis of GO and KEGG pathways, $p < 0.05$ was considered requisite to achieve significant enrichment.

Dual-luciferase reporter assays

The luciferase system was established through the application of the Dual Luciferase Reporter Gene Assay Kit (Beyotime, RG027). The vector comprising loaded promoters (wild type or mutant type) of RBM47 or LINC00862 was co-transfected with another effector once the HCCLM3 cells had covered 75% of the bottom of the 24-well culture plate. The vectors were mixed with Lipofectamine 2000 (1:1) prior to transfection at room temperature. Following 48 hours of culturing, the cells were lysed, centrifuged, and the supernatant was sequentially assessed utilizing firefly and renilla luciferase detection solution. The mean of the ratio of firefly luciferase and renilla luciferase was established as normalization for quantitative comparison.

RNA immunoprecipitation (RIP) analysis

RIP was undertaken via employment of the Magna RIP™ RNA-Binding Protein Immunoprecipitation Kit (Merck, 17-700) in accordance with the manufacturer's protocol. In summary, magnetic beads were suspended, and the antibody was added to RIP Wash Buffer, with a 30-minute incubation period at room temperature ensuing. HCCLM3 cells were lysed via the use of RIP Lysis Buffer, undergoing a 5-minute incubation on ice. The beads-antibody complex was then incubated with the RIP Immunoprecipitation Buffer and the supernatant of cell lysate overnight at 4°C. Following centrifugation and washing, the beads-antibody complex was re-suspended by Proteinase K Buffer with a 30-minute incubation period at 55°C. Following incubation with Salt Solutions and Precipitate Enhancer, RNA was eluted for subsequent PCR. IgG expression in PCR was established as 1, and quantitative comparison was normalized to Input, which was established as an internal reference.

Chromatin immunoprecipitation (ChIP) analysis

HCCLM3 cells were incubated in culture medium containing 1% formaldehyde

for crosslinking for a duration of 10 minutes. Crosslinking was subsequently terminated with 0.125M glycine for a further 5-minute incubation period. The cells were then lysed in ChIP lysis buffer containing 50mM HPES (Sinopharm, SH614702) and 0.1% SDS (Sinopharm, 30166428) on ice for a duration of 20 minutes. Sonication was applied for 10 minutes and the supernatant was taken after centrifugation. The products of sonication were incubated with antibodies at 4℃ overnight for immunoprecipitation of CHD5 or Flag targeting chromatin fragments. Pierce™ Protein G agarose (Thermo Scientific, 20421) was then mixed for a further 2-hour incubation period at 4℃. DNA was eluted utilizing elution buffer (1% SDS, 100mM NaHCO3) and purified via RNase A (CWBIO, CW0601S) and Proteinase K (Beyotime, ST533) before undergoing subsequent PCR.

Chromatin isolation by RNA purification (ChIRP) analysis

The ChIRP methodology was executed according to a previously established protocol [8]. In essence, a suspension containing 1×10^7 cells/ml was exposed to 3% paraformaldehyde at ambient temperature for 30 minutes to establish a cross-link. The formaldehyde is quenched using 1.25 M glycine, and the cells were lysed using a Lysis Buffer. The lysate was subjected to sonication in a 4℃ water bath for 4 hours, followed by the utilization of the FastPure Gel DNA Extraction Mini Kit (Vazyme, DC301) to carry out the purification of DNA. The purified DNA was then hybridized with an RNA probe pool [9] at a concentration of 100 pmol per ml of chromatin in 2 ml of Hybridization Buffer. Post hybridization, 100μL of magnetic beads were added to each tube, and the mixture was incubated at 37℃ with shaking 30 minutes. Then, the beads were washed with 1 ml of wash buffer, and the eluted sample was split into 100μL for protein detection and 900μL for DNA fraction. For protein detection, the sample was pre-treated with 40μL of RIPA and 10μL of loading buffer and subjected to CHD5 (Proteintech, 61684, 1:1000) protein blotting using the same method as above. Additionally, 150μL of DNA elution buffer containing a concentration of 10 μL/mg of RNase A and RNase H were added to each sample for DNA extraction, and this step was repeated twice with shaking at 37℃ for 30 minutes. Finally, subsequent PCR was carried out using two pairs of primers, and the input was used as an internal

control, while the enriched level by lacZ was normalized to 1.

DNA Pulldown assay

For DNA pulldown, 400pmol DNA probes (p-RBM47 or p-LINC00862) were incubated with 50μL washed streptavidin magnetic beads and 500μL nucleic dilution buffer (50mM Tris-Hcl, pH 7.5, 5mM EDTA, 0.05% Tween-20) for a period of 90 minutes at room temperature. Following a 1-minute centrifugation period at room temperature, the supernatant was aspirated, and 500μL of wash buffer (50mM Tris-HCl, pH 7.5, 150mM NaCl, 10% glycerol, 10mM sucrose, 5 mM EDTA, 0.1% TritonX-100) was added for purification. A 1mL reaction system comprising of 5mg total protein and protein dilution buffer (50mM Tris-HCl, pH 7.5, 0.1mM EDTA, 0.1% Tween-20, 0.5% KCl, 1mM $MgCl_2$, 10% glycerol) was mixed and incubated overnight at 4°C. Following the washing and collection of magnetic beads on a magnetic rack, the magnetic beads were boiled with the 2 × protein loading buffer for a period of 5 minutes, which primed them for subsequent Western blotting or mass spectrometry identification. Biotin-NC probes were applied as a negative control.

RNA pulldown assay

RNA pulldown was achieved through utilization of the Pierce™ Magnetic RNA-Protein Pull-Down Kit (ThermoFisher, 20164Y), following the manufacturer's protocol. The generation of full-length and sequence-deletion probes for LINC00862 necessitates *in vitro* transcription from the corresponding vectors using the T7 High Yield RNA Transcription Kit (Vazyme, TR101-01) prior to their utilization in pure RNA extraction and biotin labeling. In essence, 50μL streptavidin magnetic beads were washed via 20mM Tris (pH 7.5), before being incubated with an equal volume of 1×RNA Capture Buffer (20mM Tris, pH 7.5, 1M NaCl, 1mM EDTA) and 50pmol of biotin-labeled LINC00862 probe for a duration of 15 minutes at room temperature. After another washing period with 20mM Tris, the magnetic beads were incubated in a 100μL reaction system comprising 1×RNA Binding Buffer (0.02M Tris, pH 7.5, 0.05M NaCl, 2mM MgCl2, 0.1% Tween™-20 Detergent), 50% glycerol, lysate (protein conc. > 2mg/mL) and nuclease-free water at 4°C for a duration of 60 minutes. Ultimately, the streptavidin magnetic beads were washed with Wash Buffer (20mM

Tris, pH 7.5, 10mM NaCl, 0.1% Tween-20 Detergent), and were eluted for subsequent Western blotting or mass spectrometry.

HPLC-MS/MS

The proteins obtained from the pulldown assay were enzymatically hydrolyzed by means of incubation with dithiothreitol (Amresco, M109-5G), iodoacetamide (Amresco, M216-30G) and trypsin (Promega, V5280). Following enzyme digestion, the products were eluted with 70% acetonitrile elution (J.T.Baker, 34851) via the utilization of the C18 column in order to effectuate desalination. The aforementioned Q Exactive^{TM} HF Mass Spectrometer was utilized for MS data collection, while the peptide segment matching was executed by means of Proteome Discoverer software (V2.4). As previously mentioned, the unprocessed data was also submitted to iProX. The matched proteins sourced from the products that were specifically pulled by the LINC00862 probe or the RBM47 promoter probe would be ranked utilizing the score of SEQUEST High-Throughput (Sequest HT). Proteins that evinced missing normalized abundance would be excluded from further analysis. A high rating in terms of protein confidence would be conferred onto entries that exhibited FDR < 0.0001. The GO and KEGG enrichment analysis would be executed based on identified proteins that were specifically pulled by RBM47 promoter or LINC00862. Common proteins matched by the two probes would be deemed potential interacting regulators.

Triplex pulldown assay

A biotin-labeled wild-type double-stranded DNA (dsDNA) probe comprising of putative binding sites of RBM47 promoter (-1491/-1442), along with a corresponding dsDNA probe containing a mutant binding locus were prepared for triplex pulldown. 50μL streptavidin magnetic beads and 500μL nucleic dilution buffer, dissolving 500pmol of the aforementioned biotin-labeled probe, were incubated for a duration of 90 minutes at room temperature. Following centrifugation and removal of the supernatant, magnetic bead probes that failed to bind were aspirated before hybridizing with RNAs. 30μL of extracted total RNA (> 0.4 μg/μL) was added to hybridize with the probe-bead complex mixed with 10μL of the aforementioned

1×RNA Binding Buffer and 30μL of 50% glycerol for a period of 60 minutes at 4 ℃. All RNA binding with the dsDNA probe would be eluted for subsequent q-PCR. Both the empty bead and biotin-NC were used as negative controls with the empty bead being normalized to 1. The quantitative Ct value of input would be computed as an internal reference.

Electrophoretic mobility shift assay (EMSA)

EMSA was employed to further explore the formation of a triplex between LINC00862 and the RBM47 promoter. Initially, a streptavidin-labeled dsDNA probe containing a putative binding site was pre-treated at 93°C for 3 minutes and followed by a 10-minute incubation at 16°C. After that, a hybridization solution composed of 10mM Tris-HCl, 50mM NaCl, 10mM MgCl2, pH 7.4 was used to incubate the dsDNA probe (200nM) and the monomeric RNA segment of LINC00862 (50nM), which contained two putative binding sites, at 60°C for 1 hour. In the RNaseH group, an additional 2μL RNaseH (Merck, 10786357001) was added and incubated at 37°C for 1 hour. The mixture then underwent polyacrylamide-gel electrophoresis (1 hour at 60V) followed by horizontal electrophoresis to a nylon membrane (30 minutes at 100mA). The nylon membrane was further subjected to 40 minutes of incubation with a blocking solution (Beyotime, GS009B) at room temperature. Afterwards, 5μL of Streptavidin-HRP Conjugate (Proteintech, SA00001-0) were mixed into the blocking solution for an additional 20 minutes of incubation. Finally, the ECL Luminescent Detection Kit (Vigorous, P004) was employed for visualization and detection during the blotting process.

Supplementary references

[1] Parkhomchuk D, Borodina T, Amstislavskiy V, Banaru M, Hallen L, Krobitsch S, et al. Transcriptome analysis by strand-specific sequencing of complementary DNA. Nucleic Acids Res 2009; 37(18): e123.

[2] Barrett T, Wilhite SE, Ledoux P, Evangelista C, Kim IF, Tomashevsky M, et al. NCBI GEO: archive for functional genomics data sets--update. Nucleic Acids Res

2013; 41(Database issue): D991-5.

[3] Cox J, Mann M. MaxQuant enables high peptide identification rates, individualized p.p.b.-range mass accuracies and proteome-wide protein quantification. Nat Biotechnol 2008; 26(12): 1367-72.

[4] Cox J, Hein MY, Luber CA, Paron I, Nagaraj N, Mann M. Accurate proteome-wide label-free quantification by delayed normalization and maximal peptide ratio extraction, termed MaxLFQ. Mol Cell Proteomics 2014; 13(9): 2513-26.

[5] Lazar C, Gatto L, Ferro M, Bruley C, Burger T. Accounting for the Multiple Natures of Missing Values in Label-Free Quantitative Proteomics Data Sets to Compare Imputation Strategies. J Proteome Res 2016; 15(4): 1116-25.

[6] Ma J, Chen T, Wu S, Yang C, Bai M, Shu K, et al. iProX: an integrated proteome resource. Nucleic Acids Res 2019; 47(D1): D1211-D1217.

[7] Chen T, Ma J, Liu Y, Chen Z, Xiao N, Lu Y, et al. iProX in 2021: connecting proteomics data sharing with big data. Nucleic Acids Res 2022; 50(D1): D1522-D1527.

[8] Chu C, Quinn J, Chang HY. Chromatin isolation by RNA purification (ChIRP). J Vis Exp 2012; (61): 3912.

[9] Raj A, Bogaard P, Rifkin SA, Oudenaarden A, Tyagi S. Imaging individual mRNA molecules using multiple singly labeled probes. Nat Methods 2008; 5(10): 877-9.

Supplementary figures and legends

Figure S1

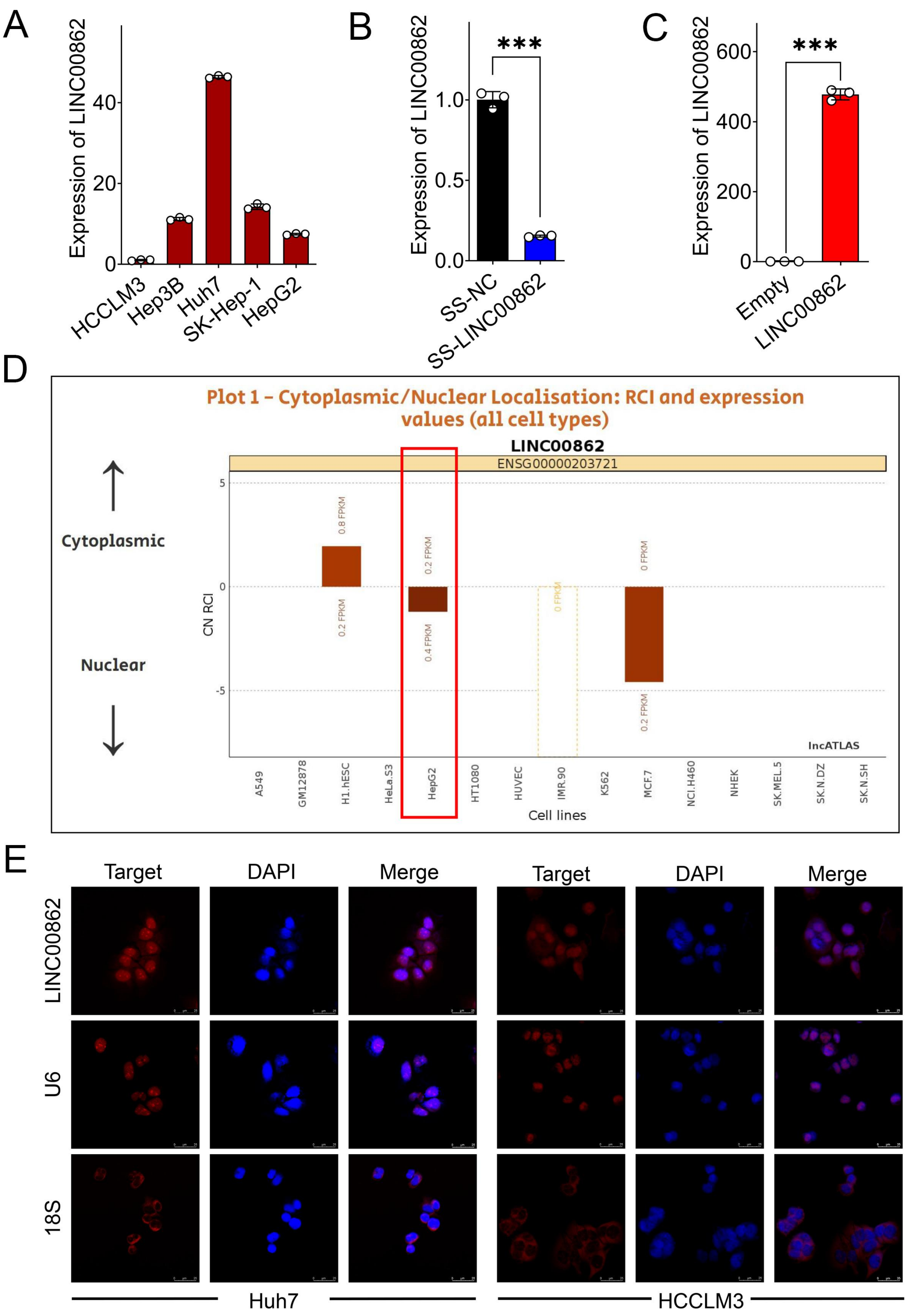

Figure S1. The characteristics of LINC00862 in hepatoma cells.

(A) The expression profile of LINC00862 in diverse hepatoma cell lines. (B) The

effectiveness of SS targeted to LINC00862 in silencing its expression in Huh7 cells, as assessed via qRT-PCR. (C) The upregulation of LINC00862 after transfection of a pcDNA3.1 vector loaded with LINC00862 in HCCLM3 cells is displayed, as measured by qRT-PCR. (D) An online lncATLAS database is utilized to predict the subcellular localization of LINC00862 in HepG2 cells, with the red box outlining its expression in hepatoma cells. (E) The subcellular localization of LINC00862 in both Huh7 and HCCLM3 cells using RNA FISH, with U6 and 18S as references for the nuclear and cytoplasmic compartments. White bar = 25 μm. SS, smart silencer, ***P<0.001.

Figure S2

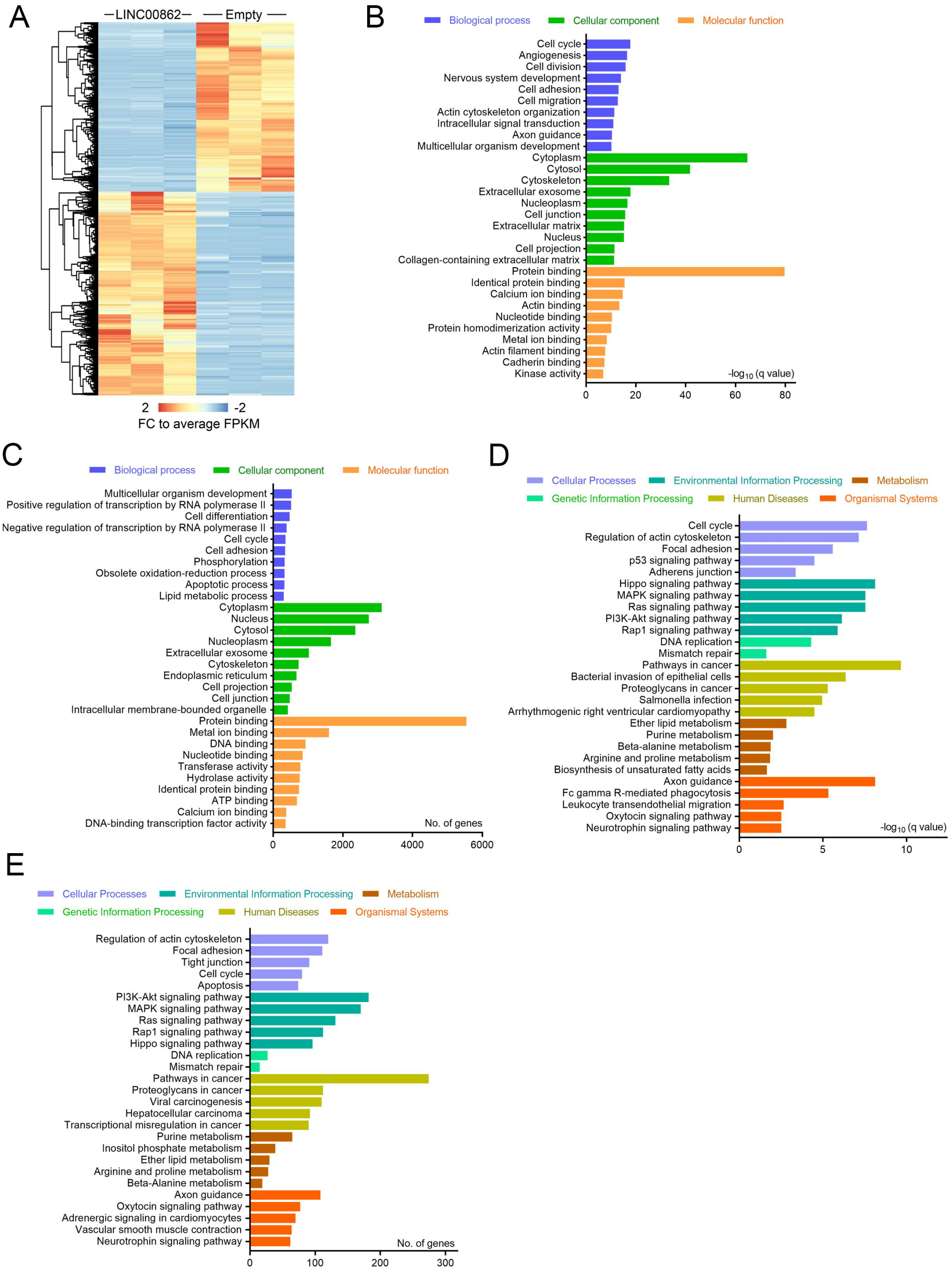

Figure S2. The results of RNA-sequencing following the overexpression of LINC00862 in cells showcase the alterations of genes and associated pathways. (A) The heatmap exhibiting the genes displaying significant differential levels. (B, C) Gene Ontology (GO) analysis is presented, with panels depicting the top 10 most significant and enriched genes in three GO categories: molecular function, cellular

component, and biological process. (D, E) KEGG pathway analysis is showcased, with featuring the top 5 pathways ranked by significance and enrichment among six categories, respectively. Categories with fewer than 5 enriched pathways indicate that the $P<0.05$ threshold was not met for these specific pathways.

Figure S3

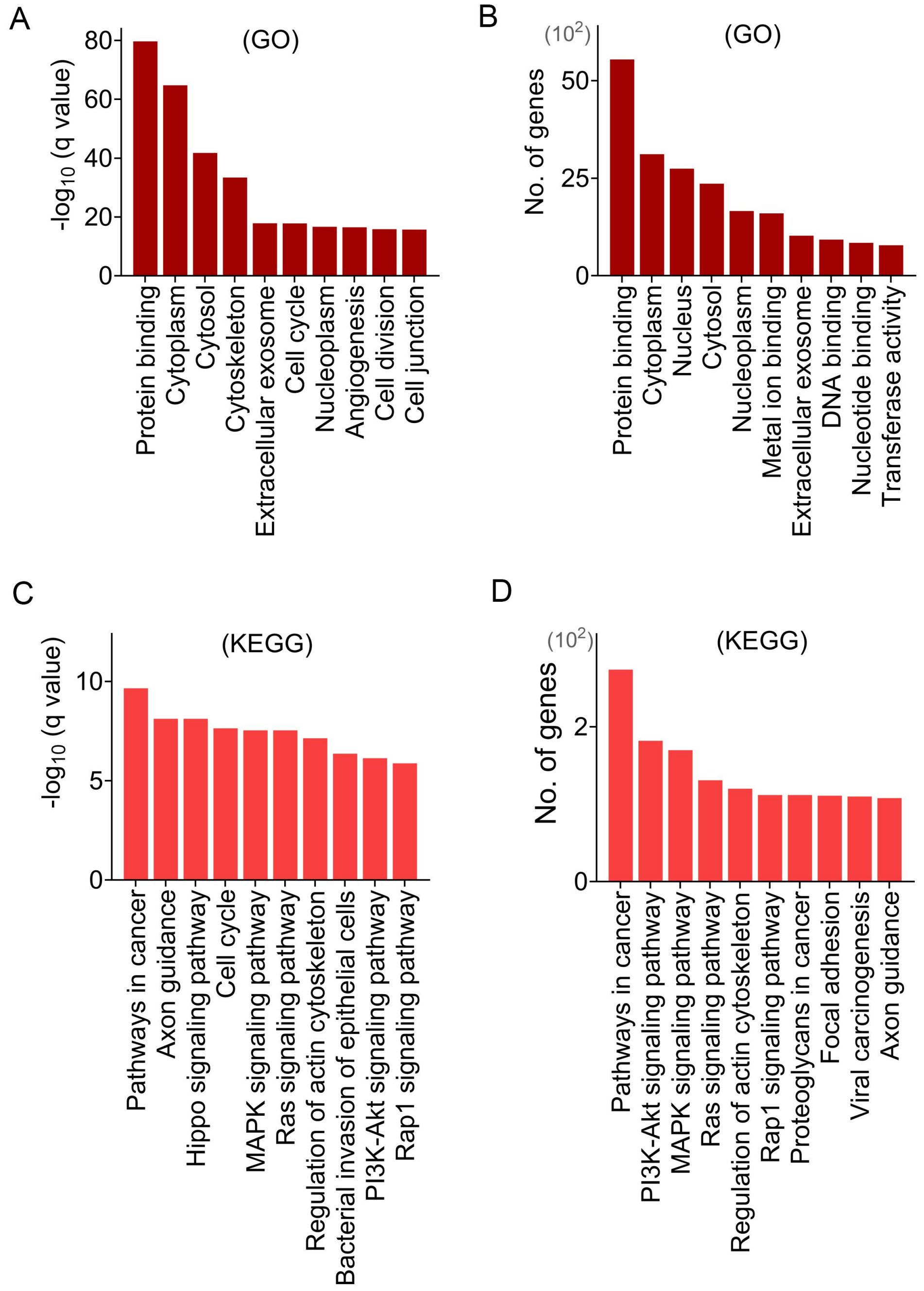

Figure S3. The summary of the top 10 items identified through GO and KEGG analysis based on RNA sequencing.

GO analysis was used to identify the top 10 items with (A) the lowest q values and (B) greatest gene enrichment in RNA sequencing. KEGG analysis of RNA sequencing was conducted, and the top 10 pathways with (C) the lowest q values and (D) most enriched genes are presented.

Figure S4

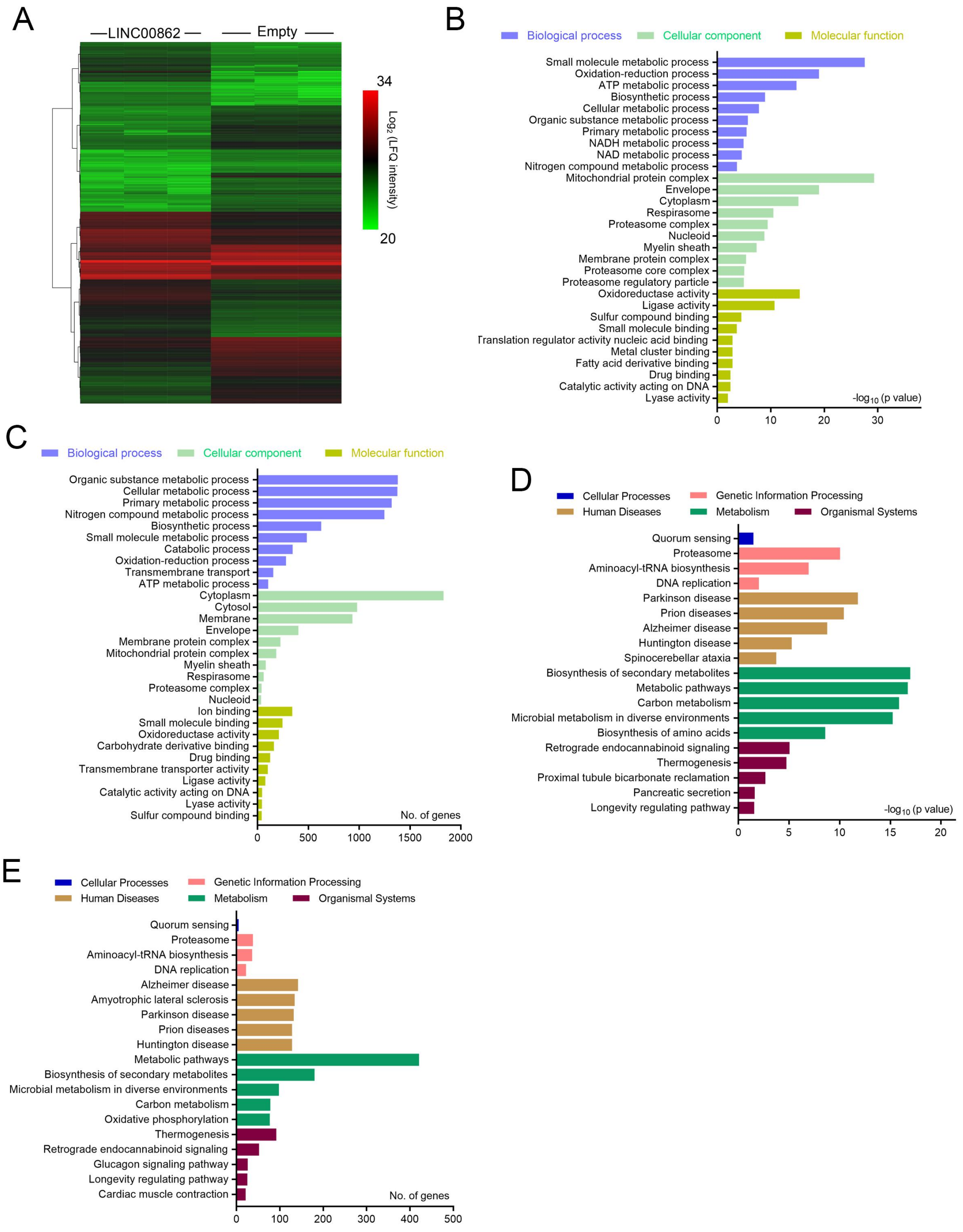

Figure S4. The characterization of protein expression after LINC00862 overexpression is depicted using quantitative proteomics.

(A)The heatmap revealing the proteins with significant fold changes. (B, C) GO analysis is presented, with showing the top 10 most significant and enriched genes in three GO categories: molecular function, cellular component, and biological process. (D, E) KEGG pathway analysis is depicted, with featuring the top 5 pathways ranked by significance and enrichment among 5 categories, respectively. Categories with

fewer than 5 enriched pathways indicate that the $P<0.05$ threshold was not met. Notably, the category of Environmental Information Processing did not exhibit any pathway enrichments with $P<0.05$ in quantitative proteomics.

Figure S5

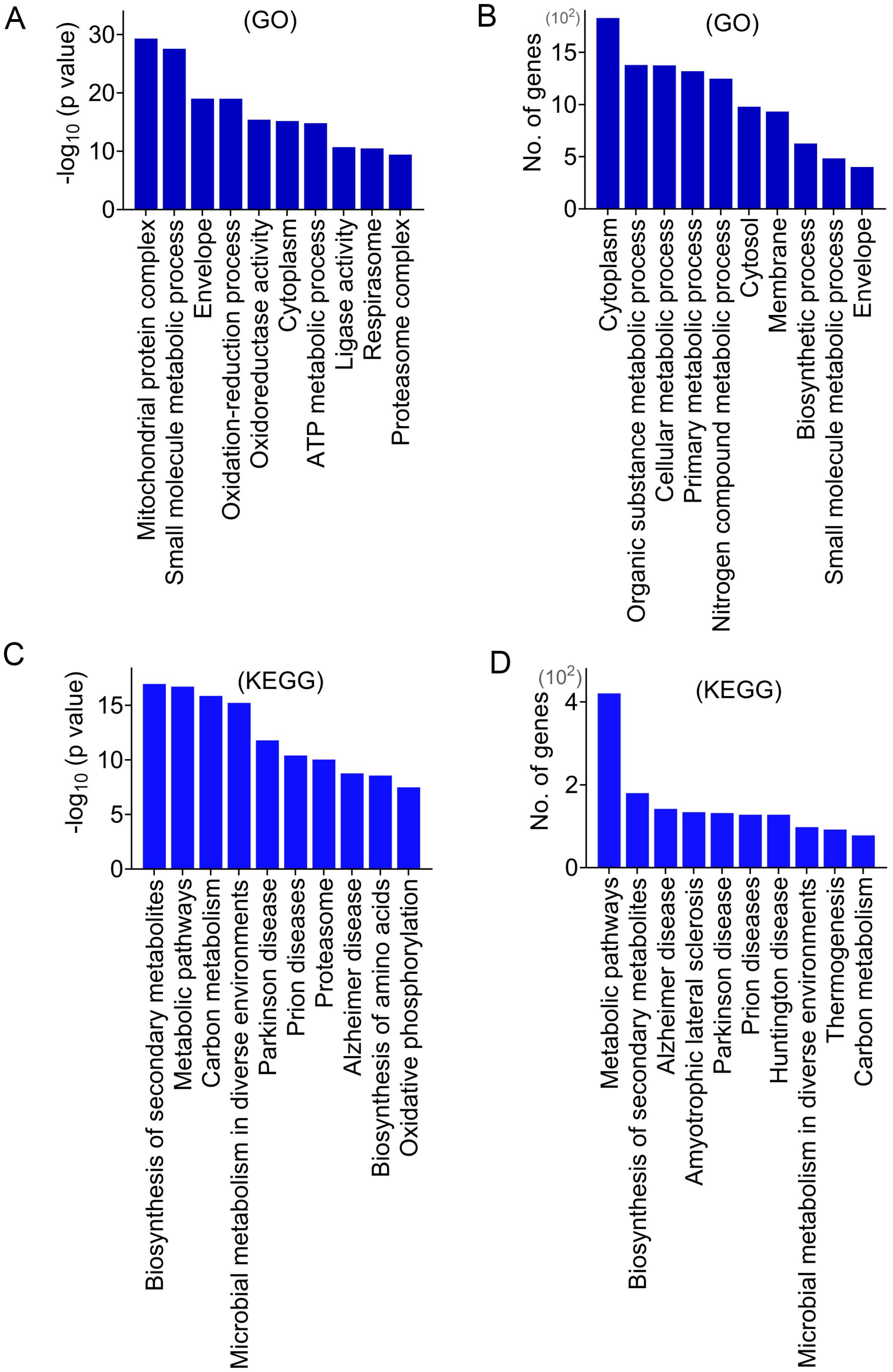

Figure S5. The top 10 items ranked according to GO and KEGG analysis based on quantitative proteomics data.

(A, B) GO analysis of quantitative proteomics data revealed the top 10 most

significant genes with the lowest p values and highest gene enrichment. (C, D) KEGG analysis was performed on the quantitative proteomics data, and the top 10 pathways with the lowest p values and most enriched genes are also illustrated.

Figure S6

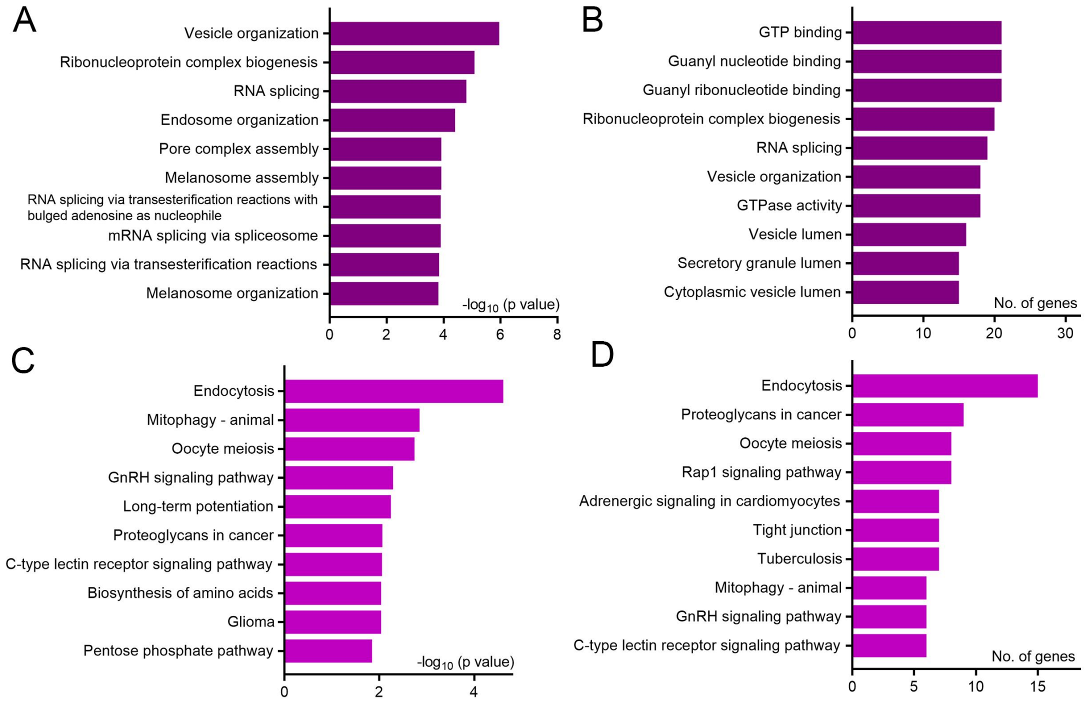

Figure S6. The results of an enrichment analysis of proteins that interact specifically with the RBM47 promoter.

(A, B) The analysis of GO enrichment identified the top 10 GO terms exhibiting the lowest p-values and the most enriched genes, highlighting their significant biological functions. (C, D) The KEGG analysis yielded the top 10 enriched pathways with the lowest p-values and the most significantly enriched genes.

Figure S7

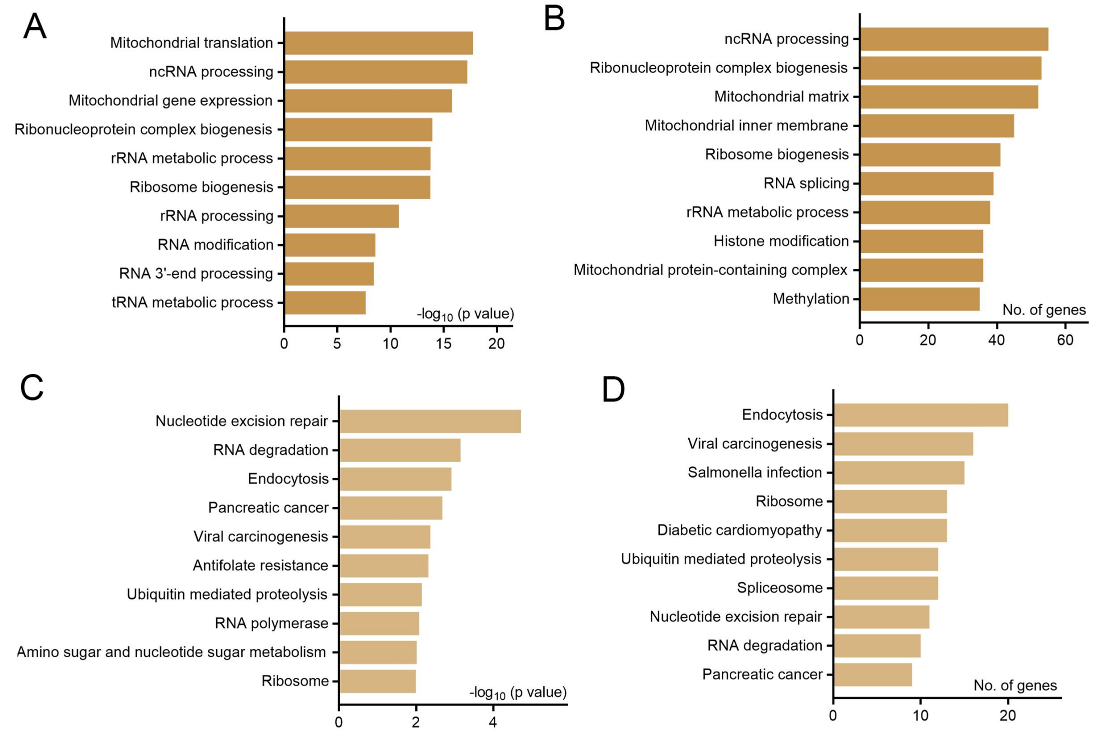

Figure S7. The enrichment analysis was conducted on proteins that specifically interacted with LINC00862.

(A, B) Gene Ontology analysis identified the top 10 terms exhibiting the lowest p-values and the most enriched genes. (C, D) The top 10 enriched pathways with the most significantly enriched genes and lowest p-values were identified based on KEGG analysis.

Figure S8

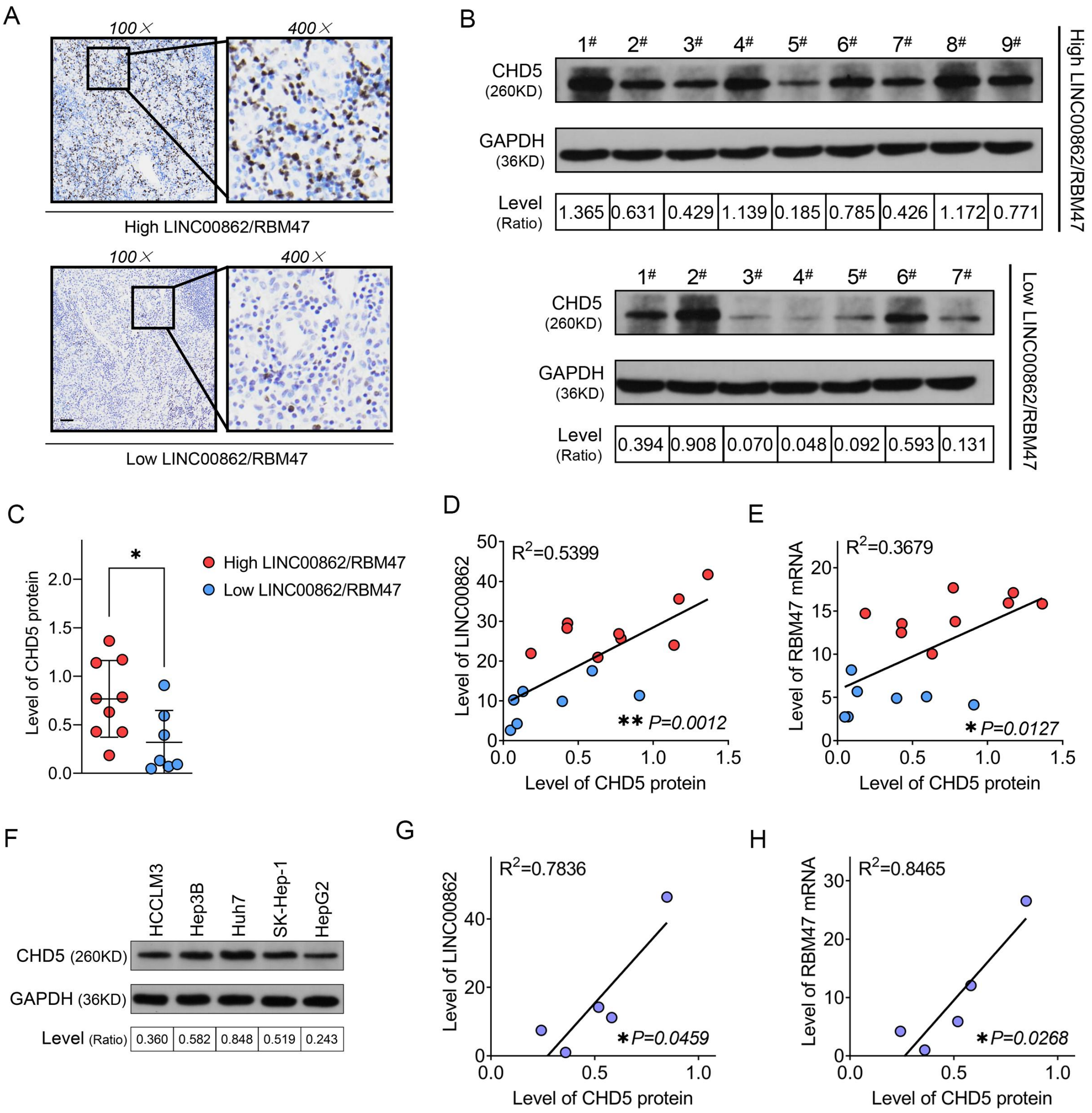

Figure S8. The expression of CHD5 protein demonstrates a positive correlation with the RNA levels of LINC00862 and RBM47 in HCC.

(A) IHC staining and (B) western blotting were employed to evaluate CHD5 protein levels in HCC tissues from low and high LINC00862/RBM47 expression groups. (C) Comparative assessment of CHD5 protein levels in HCC tissue samples with low versus high LINC00862/RBM47 expression. The correlation of CHD5 protein expression with (D) LINC00862 and (E) RBM47 mRNA levels is illustrated in HCC tissues, with red and blue points representing high and low expressions of LINC00862 and RBM47, respectively. (F) Western blotting and quantitative analysis were performed to assess the expression levels of CHD5 protein in five distinct hepatoma

cell lines. The correlation between CHD5 protein expression and (G) LINC00862 and (H) RBM47 mRNA levels was analyzed in five identical hepatoma cell lines.

Figure S9

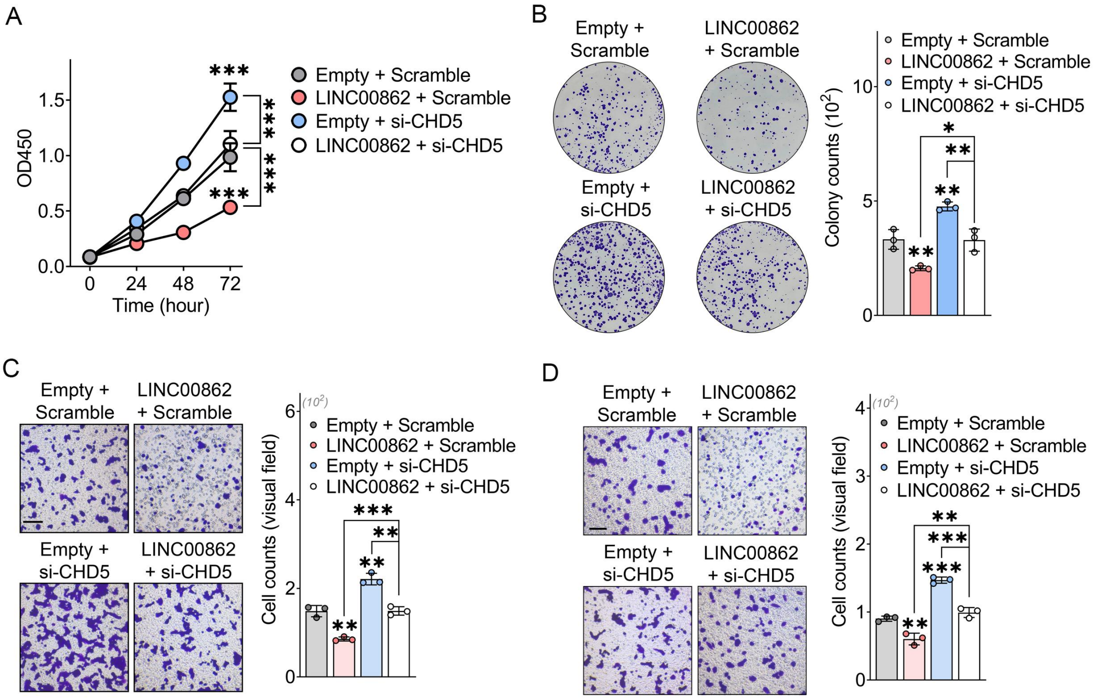

Figure S9. Impact of CHD5 on LINC00862-mediated suppression of HCC progression.

Following the overexpression of LINC00862 and knockdown of CHD5 in HCCLM3 cells, (A) CCK-8 assays and (B) colony formation assays were conducted to assess the cellular proliferation and growth capacities. Additionally, transwell assays were performed to evaluate (C) migration and (D) invasion abilities of the cells.

Figure S10

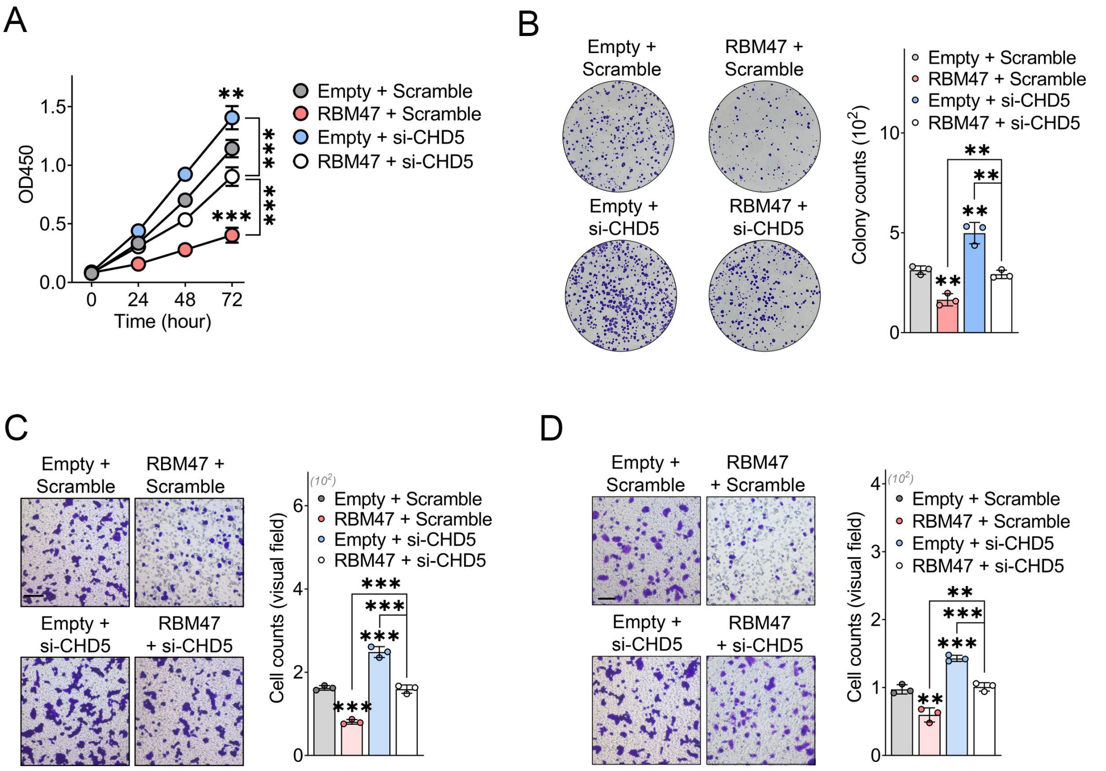

Figure S10. Collaborative inhibition of HCC progression by CHD5 and RBM47. In HCCLM3 cells wherein RBM47 was overexpressed and CHD5 was knocked down, (A) CCK-8 assays, (B) colony formation assays, and (C-D) transwell assays were performed to evaluate cellular proliferation, growth, migration, and invasion capacities.

Figure S11

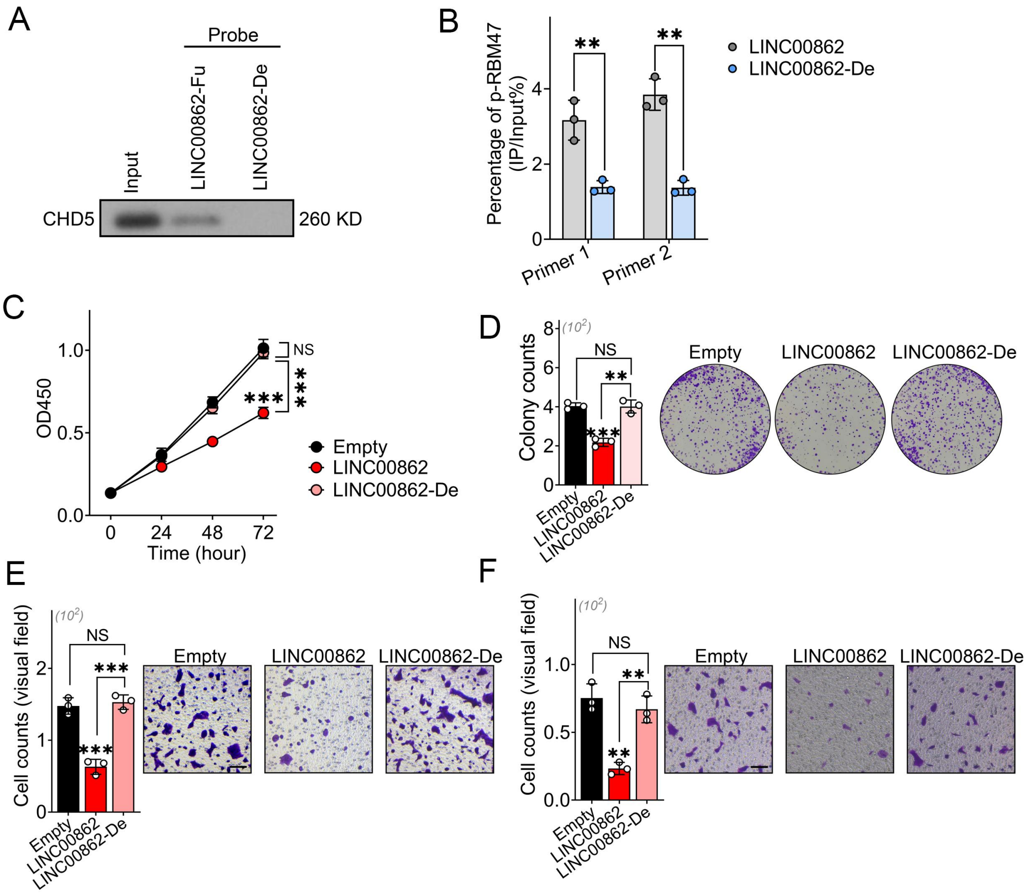

Figure S11. The specific binding region of CHD5 on LINC00862 is crucial for the interaction between CHD5 and the RBM47 promoter, as well as for the *in vitro* tumor-suppressive function of LINC00862.

(A) Western blotting was employed to assess the binding of CHD5 to both LINC00862 full-length probe and LINC00862 sequence-deletion probe following RNA pulldown. (B) Following the overexpression of full-length LINC00862 or sequence-deletion LINC00862 in HCCLM3 cells, the interaction between CHD5 and the RBM47 promoter was assessed using ChIP-PCR. After the overexpression of full-length LINC00862 or sequence-deletion LINC00862 in HCCLM3 cells, the proliferation and colonizing ability were respectively assessed using the (C) CCK-8 assay and (D) colony formation assay. After exogenously increasing the expression levels of full-length LINC00862 or sequence-deletion LINC00862 in HCCLM3 cells, the *in vitro* (E) migration and (F) invasion capabilities were assessed using the transwell assay. Fu, full-length; De, deletion.

Figure S12

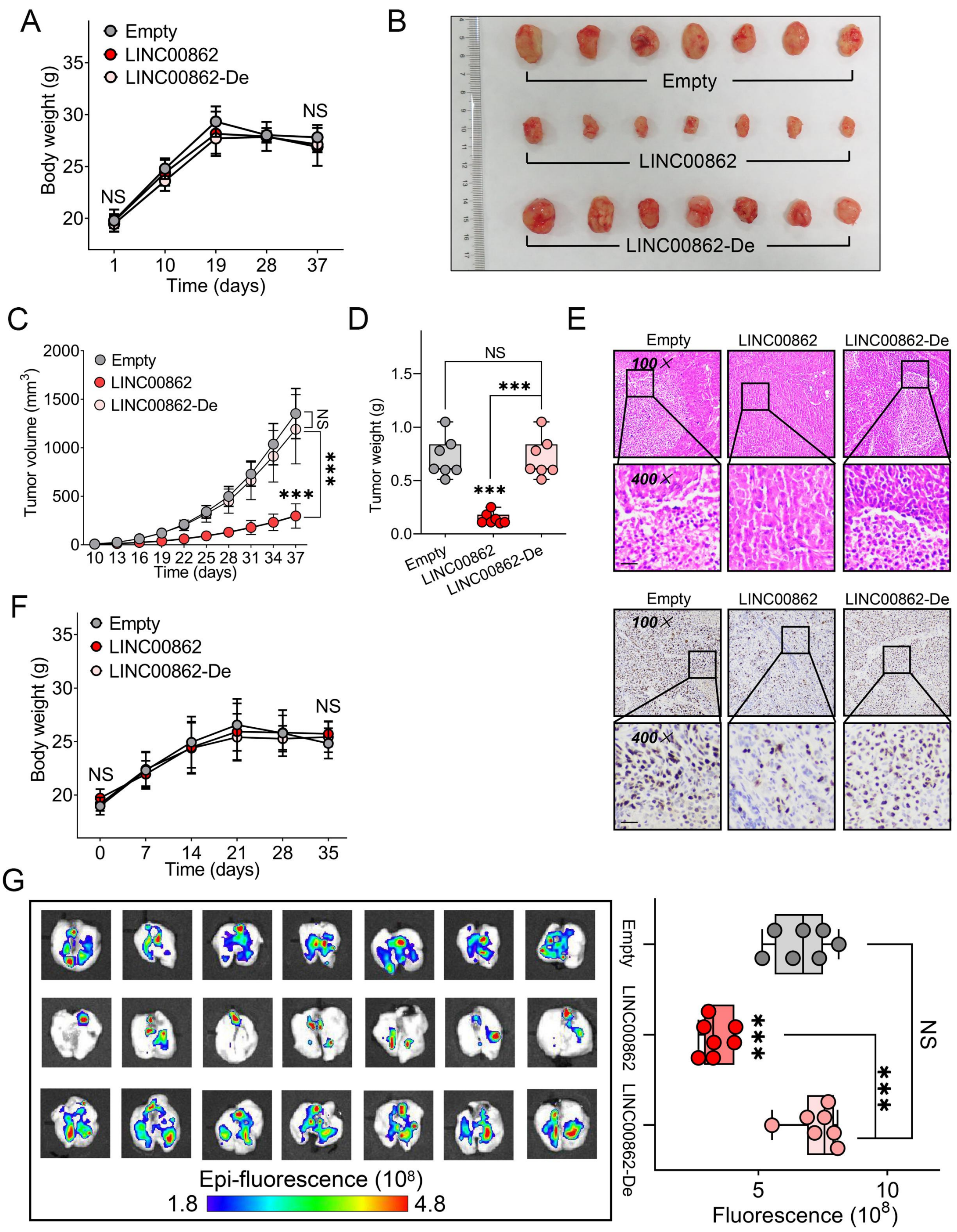

Figure S12. The specific binding region of CHD5 on LINC00862 is critical for the *in vivo* tumor-suppressive function of LINC00862 (A) Body weight measurements of nude mice were recorded from the time of subcutaneous tumor implantation until sacrifice. (B) Representative images of xenograft tumors generated from HCCLM3 cells stably transfected with either LINC00862 or LINC00862-De. (C) Growth curves

and (D) tumor weights were evaluated. (E) Histological examination of xenograft tumors stably expressing LINC00862 and LINC00862-De was performed, including HE staining (upper panel) and immunohistochemical analysis for the proliferation marker Ki-67 (lower panel), with scale bar = 50 μm. (F) Weight changes of nude mice were monitored in three groups following the establishment of the pulmonary metastasis model. (G) In vivo imaging of lung tissues (left) and quantitative fluorescence analysis (right) were conducted after tail-vein injection of HCCLM3 cells stably expressing LINC00862 and LINC00862-De.

Supplementary tables

Table S1. The primers implemented in the present investigation.

Gene	Sequence
LINC00862-F	CAAGCCAAGGAGCACCAACAG
LINC00862-R	CAGAAGTCCCAAGTCCCAAATCC
pre-LINC00862-E4-I4-F	AACTTTCCCCAGCATCAGCC
pre-LINC00862-E4-I4-R	CTAATTTCGTAACCTGCGGACA
pre-LINC00862-I4-E5-F	ACCGTTACCGAACATACAGTCT
pre-LINC00862-I4-E5-R	AGCCATGATAACTGGATGCAA
U6-F	GCTCTCGCATCGCAGCA
U6-R	CTCGGCTAGCGCTACTC
RBM47-F	ATCAGCAATCCTTGGCTCAC
RBM47-R	CCTTGGGATTCCTCTGTTCA
p-RBM47-F1 for ChIP	CAAGCAGGAGTTTTCCCTGA
p-RBM47-R1 for ChIP	GTGGCAGGCACCTGTAATTC
p-RBM47-F2 for ChIP	TGATACCCCTTCAGCCTTTG
p-RBM47-R2 for ChIP	ATTTTGTCCCCATTTTGCAG
p-LINC00862-F1 for ChIP	TGTGTAACCACGGACCTTGA
p-LINC00862-R1 for ChIP	CCCGACGACAGCTTTCTAAG
p-LINC00862-F2 for ChIP	TAACCCTGCTTTCCACCATC
p-LINC00862-R2 for ChIP	GGCTCAACCAGTTCAAAAGC
p-RBM47-F1 for ChIRP	CATCCGCCTCACAGTAGCAA
p-RBM47-R1 for ChIRP	GTCCCCCAATCTCTGCCATC
p-RBM47-F2 for ChIRP	AACCCAGATGTCCGCAATCC
p-RBM47-R2 for ChIRP	TGAATGACACTGCAACCCGA

Table S2. The sequences of NC probes and ChIRP probes.

Probe	Sequence
DNA NC probe	TGAGTAAAGGAGAAGAACTTTTCACTGGAGTTGTCCCAATTCTTG TTGAA
RNA NC probe	UUGUGCCCAUUAACAUCACCAUCUAA
Probe pool for ChIRP	LINC00862 probes<br>Probe-1 GTGACAGCCTACAGACTGCTATT<br>Probe-2 CTCGTGCTGACATCATCGGA<br>Probe-3 TCCTTCCCTCCTTCGTGTCT<br><br>LacZ probes<br>Probe-1 CACCCTGCCATAAAGAAACT<br>Probe-2 GTAGTTCAGGCAGTTCAATC<br>Probe-3 GTTGTATTGTAACAGTGGCC

Table S3. The 20 highest-ranked Sequest HT protein interactors with specificity for the RBM47 promoter.

Accession	Gene Name	Abundances (Normalized)	Coverage [%]	Peptides	MW [kDa]	Score of Sequest HT
P68366	TUBA4A	16341480.87	32	13	49.9	66.79
P31483	TIA1	66503329.38	34	12	42.9	53.72
Q562R1	ACTBL2	349248.1875	18	9	42	32.73
Q96EP5	DAZAP1	22983713.23	31	9	43.4	26.17
P57721	PCBP3	657540.3125	19	5	39.4	24.04
P40429	RPL13A	22411931.13	20	5	23.6	19.5
Q52LJ0	FAM98B	180318.1563	12	5	45.5	17.74
Q86Y46	KRT73	2046291.25	8	5	58.9	17.22
Q9UKV8	AGO2	12885917.16	12	9	97.1	14.48
Q86SE5	RALYL	17486162	4	2	32.3	11.45
Q15323	KRT31	1831098.625	8	4	47.2	10.32
P58107	EPPK1	1443079.375	3	4	555.3	10.11
Q9UKA9	PTBP2	602656.6875	6	2	57.5	10.06
Q15286	RAB35	1126214.125	28	6	23	9.55
Q9BQ04	RBM4B	662253.6875	17	6	40.1	9.4
P18085	ARF4	7460368.75	16	3	20.5	9.09
Q03135	CAV1	4363707.844	13	2	20.5	8.74
P61020	RAB5B	606232.25	16	3	23.7	8.34
Q8TDI0	CHD5	5625887	2	4	222.9	8.17
P14136	GFAP	612581.375	5	3	49.9	7.51

Table S4. The Sequest HT analysis revealed the top 20 proteins specifically captured by LINC00862.

Accession	Gene Name	Abundances (Normalized)	Coverage [%]	Peptides	MW [kDa]	Score of Sequest HT
Q16777	H2AC20	1930013430	41	8	14	209.06
P04259	KRT6B	1574690.875	38	21	60	144.11
Q15287	RNPS1	190876221.4	26	7	34.2	78.83
Q5XKE5	KRT79	216439280	14	9	57.8	75.01
Q96T58	SPEN	19030417.63	10	29	402	68.49
Q6S8J3	POTEE	2861248	9	9	121.3	64.52
P10412	H1-4	412458563.4	28	8	21.9	56.6
Q8TDI0	CHD5	1909544.5	9	15	222.9	55.46
P22090	RPS4Y1	11878813	26	8	29.4	46.52
P35241	RDX	565157.9375	17	12	68.5	45.95
Q9BRL6	SRSF8	8419928.688	15	6	32.3	43.55
P12814	ACTN1	513507.2813	12	10	103	41.31
P62979	RPS27A	208665261.5	47	7	18	37.38
Q99613	EIF3C	24155707.25	15	11	105.3	35.78
P28370	SMARCA1	9619097.469	13	15	122.5	35.47
Q5T200	ZC3H13	15701838.13	10	14	196.5	34.18
Q8IYB3	SRRM1	17679234.88	14	8	102.3	33.48
Q8NDF8	TENT4B	15472733.88	22	9	63.2	29.94
Q96QR8	PURB	6986954.125	42	9	33.2	26.5
Q9UQ16	DNM3	617002.5625	11	8	97.7	24.11
